# Groundwater microbial community structure under contrasting hydrological conditions in a sulfate-rich karst aquifer

**DOI:** 10.3389/fmicb.2026.1883366

**Published:** 2026-09-09

**Authors:** Renato Romano, Tom Morita, Francielli Peres, Rebeca Lizárraga, Lucas Padoan, Maria Nascimento, Livia Rocha, Amanda Bendia, Ivo Karmann, Vivian Pellizari

**Affiliations:** 1 Oceanographic Institute, University of São Paulo, São Paulo, Brazil; 2 Geosciences Institute, University of São Paulo, São Paulo, Brazil

**Keywords:** genome-resolved metagenomics, groundwater microbiome, hydrochemical heterogeneity, karst aquifer, redox gradients, subsurface microbiology, sulfur cycling

## Abstract

Karst aquifers represent dynamic subsurface environments where hydrological fluctuations and redox variability can strongly influence groundwater microbiomes and associated biogeochemical processes. However, microbial sulfur cycling in sulfate-rich karst aquifers remains poorly understood, particularly from a genome-resolved perspective that links hydrochemical gradients to microbial metabolic potential. Here, we investigated how hydrochemical heterogeneity and contrasting hydrological conditions relate to microbial community composition and sulfur-cycling potential in the Irecê Basin karst aquifer (Salitre Formation, northeastern Brazil), a sulfur-rich non-hydrothermal karst system. Hydrochemical analyses, 16S rRNA gene sequencing, and genome-resolved metagenomics were integrated to characterize groundwater microbial communities and evaluate relationships between hydrochemical variability and sulfur-related metabolic potential. Samples displayed significant hydrochemical heterogeneity, including remarkable variation in oxidation–reduction potential, dissolved oxygen, sulfate concentrations, and dissolved sulfide accumulation. Amplicon sequencing of 10 samples generated more than 10,000 high-quality reads each and revealed substantial variation in community composition among groundwater samples, with substantial differences among environments and contrasting hydrological conditions, highlighting pronounced spatial and habitat-level heterogeneity. A total of 185 dereplicated medium- and high-quality metagenome-assembled genomes (MAGs) were recovered from three samples, many representing potentially novel microbial lineages. M00596-associated sulfur-transformation modules were detected in MAGs from all three metagenomic samples, whereas complete SOX modules were restricted to the comparatively more oxidizing groundwaters. Several MAGs contained both M00596 and SOX modules, indicating the potential for multiple sulfur-transformation pathways within the same genomes. Overall, these findings indicate that sulfur cycling represents a central component of groundwater biogeochemistry in this sulfate-rich karst aquifer and that hydrochemical heterogeneity is associated with variation in microbial community structure and sulfur-cycling metabolic potential. Together, the combination of sulfur-rich carbonate lithologies, heterogeneous redox conditions, and diverse sulfur-cycling microbial populations highlights the potential importance of sulfur transformations in the ecological and biogeochemical functioning of the Irecê Basin karst aquifer, with possible implications for local geochemical processes.

## 1 Introduction

Karst aquifers are among the most important groundwater reservoirs on Earth and represent dynamic subsurface environments where hydrological, geological, and biogeochemical processes strongly interact (Ford et al., 2007; Shao et al., 2026). Groundwater circulation through carbonate rocks creates heterogeneous physicochemical conditions characterized by spatial and temporal variations in redox state, dissolved ions, and nutrient availability. These environments are typically oligotrophic and energy-limited, favoring microbial communities adapted to low nutrient environments, slow growth, and efficient resource utilization (Barton and Northup, 2007; Northup et al., 2011; Bendia et al., 2022). In karst aquifer systems, microbial activity can strongly influence elemental cycling and groundwater geochemistry, particularly in zones where fluctuating hydrological conditions promote transitions between oxidizing and reducing environments (Hershey and Barton, 2018; Probst et al., 2018).

Oligotrophic subsurface environments are frequently dominated by poorly characterized microbial lineages with reduced metabolic capabilities, particularly members of the Candidate Phyla Radiation and DPANN archaea (Brown et al., 2015; Dombrowski et al., 2019; Huang and Spang, 2025). These microorganisms typically possess compact genomes, incomplete biosynthetic pathways, and limited central metabolic functions, suggesting strong metabolic dependence on surrounding microbial communities (Anantharaman et al., 2018; He et al., 2021; Huang and Spang, 2025). Such genome reduction patterns are commonly associated with energy-limited environments, where metabolic streamlining and ecological interdependence may provide adaptive advantages under persistent oligotrophic conditions (Giovannoni et al., 2014). The contribution of these non-characterized lineages to groundwater biogeochemistry and sulfur cycling is still poorly understudied, despite their apparent ecological significance in subsurface ecosystems.

Sulfur cycling represents one of the major biogeochemical processes in subsurface environments, particularly in karst aquifers where redox gradients can support the coexistence of sulfate-reducing and sulfur-oxidizing microorganisms (Muyzer and Stams, 2008; Hershey and Barton, 2018). In these systems, hydrological fluctuations associated with groundwater recharge and residence time may promote transitions between oxidizing and reducing conditions, altering sulfur speciation and the distribution of microbial metabolisms (Guo et al., 2021). Under reducing conditions, sulfate-reducing microorganisms can generate dissolved sulfide, while sulfur oxidation may occur at oxic–anoxic interfaces, coupling microbial metabolism to local geochemical transformations (Muyzer and Stams, 2008).

In sulfidic and hypogenic karst systems, sulfur oxidation has been associated with sulfuric acid speleogenesis (SAS), a process in which the oxidation of hydrogen sulfide produces sulfuric acid capable of enhancing carbonate dissolution (De Waele et al., 2024). Although microbial sulfur cycling has been explored in sulfidic caves (Engel et al., 2004; Jones et al., 2015; De Waele et al., 2016; Guo et al., 2021; Turrini et al., 2024), karst groundwater systems lacking extensive open cave networks remain comparatively understudied, particularly regarding the interaction between hydrochemical variability, redox fluctuations, and microbial genome-resolved metabolic potential (De Waele et al., 2024). Seasonal recharge and groundwater residence time may significantly influence redox dynamics in karst aquifers (Gulley et al., 2011; Wynn et al., 2013), potentially restructuring microbial communities and sulfur-related metabolisms (Zhou et al., 2012; Meng et al., 2021; Guan et al., 2023).

In this study, we investigated groundwater microbial communities and their relationships with hydrochemical variability in a sulfate-rich karst aquifer located in the southern sector of the Irecê Basin, northeastern Brazil. Using integrated hydrochemical analyses, 16S rRNA gene amplicon sequencing, and genome-resolved metagenomics, we examined how microbial community structure and sulfur-related metabolic potential varied in relation to spatial hydrochemical heterogeneity, contrasting hydrological conditions, and redox variability. Although the sampling design does not allow the independent effects of hydrological period to be disentangled from spatial and hydrochemical variability, sampling across contrasting hydrological conditions and groundwater environments provides an opportunity to examine how these environmental contrasts are associated with variation in microbial community structure and metabolic potential. Specifically, we aimed to (i) characterize the taxonomic composition of groundwater microbial communities and their genomic functional potential; (ii) characterize the sulfur-cycling metabolic potential and its variation among groundwater environments; and (iii) assess how spatial hydrochemical heterogeneity and hydrological variability relate to microbial community structure and metabolic potential.

## 2 Materials and methods

### 2.1 Study area and hydrogeological setting

The karst of Southern Bacia de Irecê is located in the context of the Chapada Diamantina, which consists of a series of ranges and plateaus that serve as a division between the São Francisco River basin and the drainage to the South Atlantic Ocean. The Irecê basin consists mainly of Neoproterozoic carbonate rocks bordered by siliciclastic terrains of the Mesoproterozoic Espinhaço Supergroup (de Souza et al., 2003). The study area comprises an aquifer within the Neoproterozoic limestones of the Salitre Formation, which presents a well-developed karst system, with caves underground rivers, sinkholes, breakdown dolines, and other dissolution features becoming progressively more abundant toward the southern portion of the basin (de Queiroz Salles et al., 2018). A geological context of the groundwater sampling sites in the Irecê Basin karst system is presented in Supplementary Figure S1.

The Mesoproterozoic basement surrounding and underlying the Irecê Basin is composed of siliciclastic rocks of the Espinhaço Supergroup, subdivided into the Paraguaçu and Chapada Diamantina groups. Within the latter, the Tombador, Caboclo, and Morro do Chapéu formations comprise predominantly sandstones, conglomerates, and mudstones with local occurrences of silicified limestone (Misi, 1979; Bomfim et al., 1985; de Andrade Filho et al., 1999).

The Neoproterozoic succession overlies the siliciclastic basement through a marked erosional unconformity. At its base, the Bebedouro Formation consists of glacial diamictites containing clasts of variable sizes within a silty-clay matrix (de Souza et al., 2003). Overlying these units, the Salitre Formation comprises the predominantly carbonate succession that hosts both the regional karst aquifer and the cave systems investigated in this study. These carbonate rocks are mainly composed of calcisiltites with laminated, stratified, and locally brecciated facies (de Souza et al., 2003; de Pereira et al., 2025). Disseminated sulfide minerals, such as pyrite, galena, and sphalerite, are found with the carbonate succession and are the main sulfur source in the hydrogeochemical setting of the karst system. No primary sulfate minerals occur within the sedimentary sequence (Misi and Souto, 1975).

The carbonate surface varies from planar to gently undulated and is locally covered by Cenozoic sandy-clayey detrital deposits that fill valleys and paleoreliefs developed over the Precambrian rocks (Laureano et al., 2016). The epikarst is covered by a residual soil that is very irregular in thickness, presenting collapse and suffosion dolines, sinkholes, and karren fields. Groundwater circulation and cave development are strongly controlled by fracture systems and sub-horizontal carbonate strata, resulting in extensive branched conduit networks throughout the aquifer (Laureano et al., 2016; de Pereira et al., 2025).

The Irecê Basin is located within the semi-arid region of northeastern Brazil and presents an average annual rainfall around 600–700 mm (Villanueva et al., 2014). The main surface streams of the study area are the Santo Antônio River basin and its tributaries, such as the Água de Rega, Almas and São Jose rivers. These rivers develop as blind valleys, carving through siliciclastic terrains before entering the karst system through sinkholes and infiltration zones. Underground drainage occurs through extensive low-gradient conduit systems comprising approximately 50 km of mapped caves (Rubbioli et al., 2019). The Água de Rega River exhibits intermittent flow regimes and infiltrates directly into a collapsed sinkhole connected to the Lapa Doce system, one of the largest speleological complexes in Brazil. Subterranean drainage resurges downstream within the Pratinha Cave and adjacent spring systems distributed along the Santo Antônio River canyon, the main basal level of the aquifer. The phreatic karst system is marked by large conduits, evidenced by deep water extraction wells, some of which were sampled for analysis.

### 2.2 Sampling strategy

**FIGURE 1:**
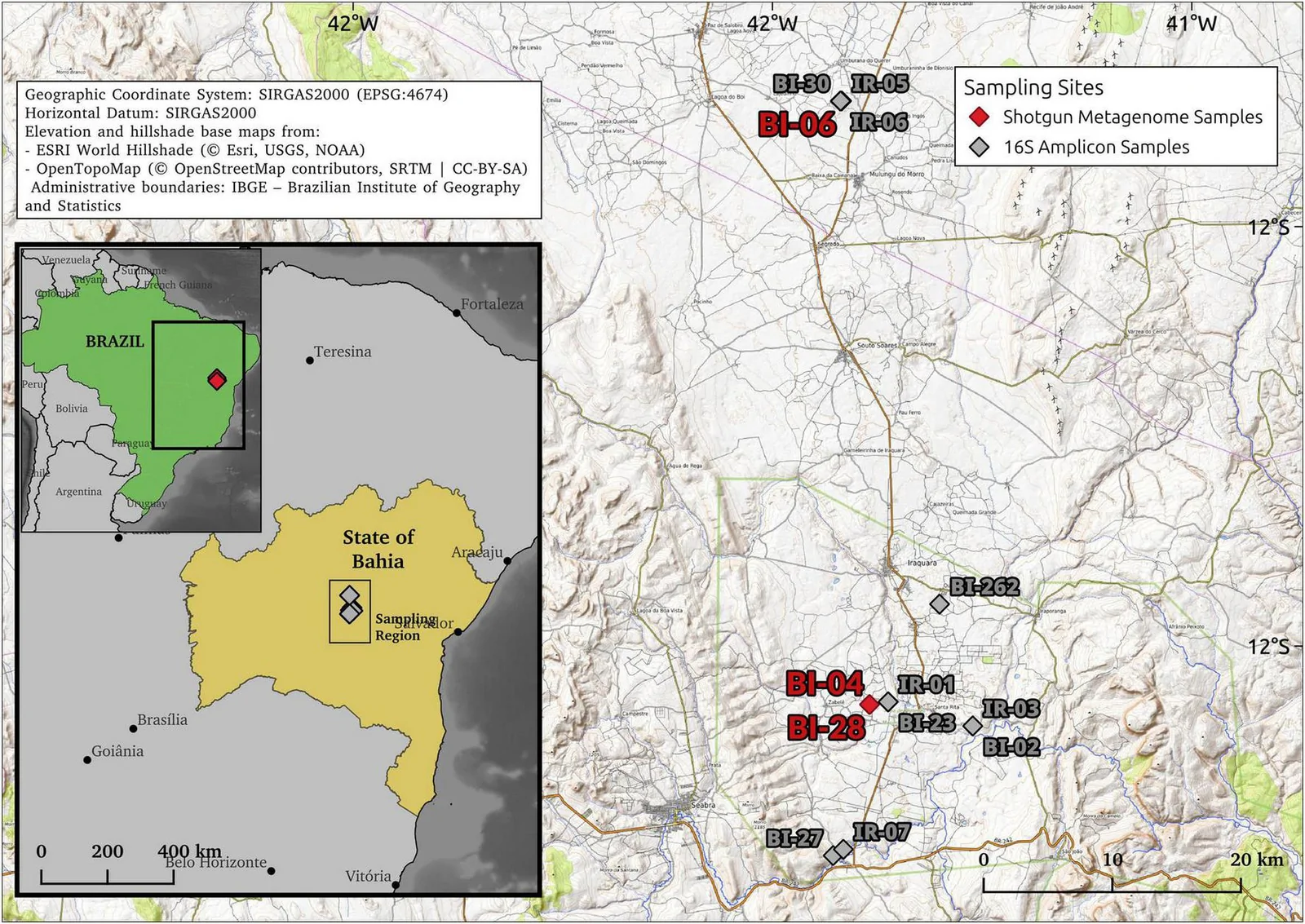
Location of groundwater sampling sites in the Irecê Basin karst aquifer system, northeastern Brazil. All groundwater sampling sites included in this study are shown. Red symbols indicate samples selected for shotgun metagenomic sequencing and MAG reconstruction, whereas the remaining sampling sites selected for 16S rRNA gene amplicon sequencing are indicated by gray symbols. Base maps were derived from ESRI World Hillshade and OpenTopoMap, with coordinates referenced to SIRGAS2000 (EPSG:4674). The map was produced using QGIS v3.34.4.

**FIGURE 2:**
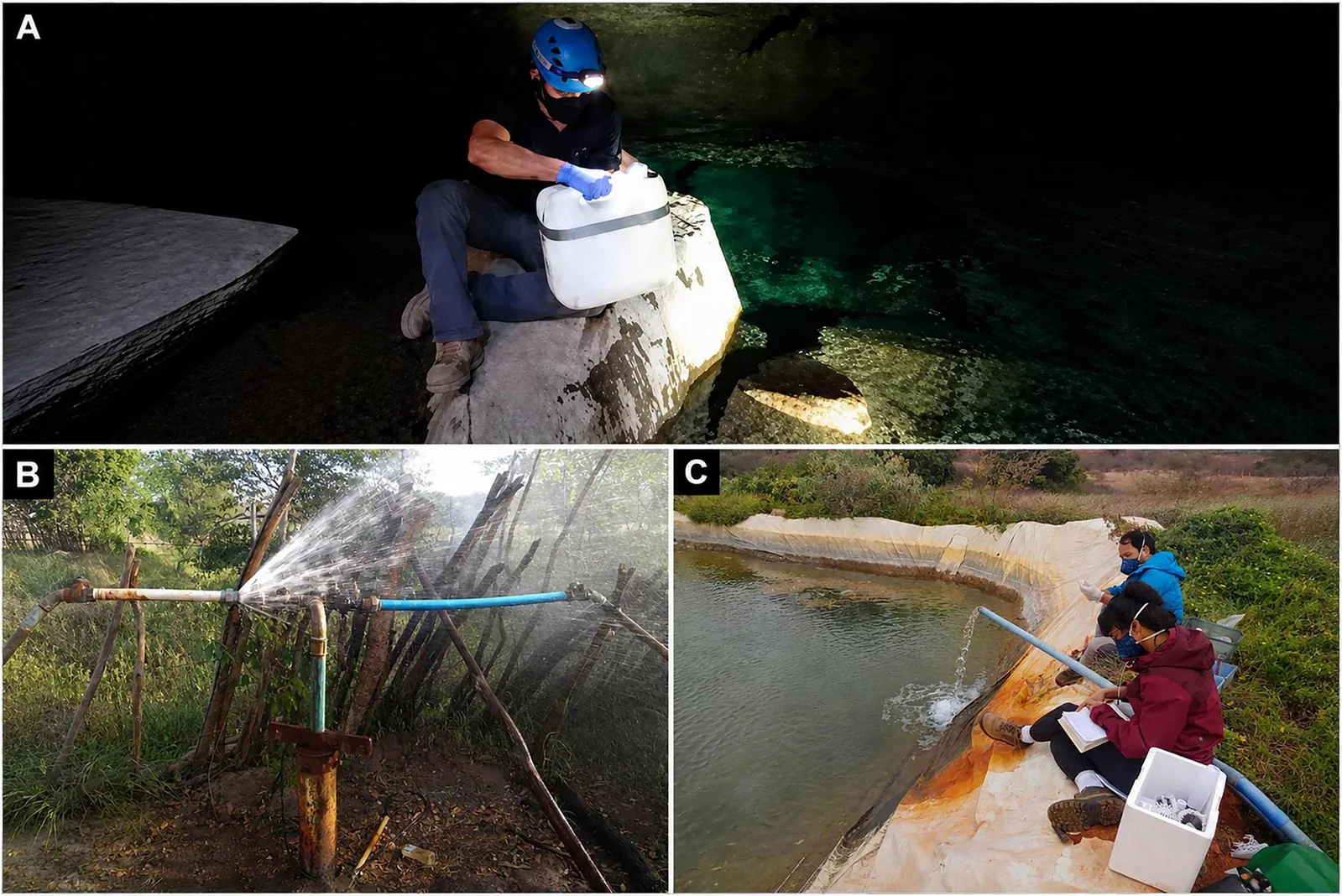
Groundwater sampling in the Irecê Basin karst aquifer system. **(A)** Water sampling inside Lapa Doce II cave, where groundwater was accessed through naturally exposed cave water under phreatic/resurgence conditions. **(B)** A partially open well located in the village of Cochó do Malheiros. **(C)** Water collection from a pipe connected to an operational well in Lagoa Preta farm, representing groundwater abstraction through established community wells used for local farming activities.

Groundwater sampling sites of the Irecê Basin karst aquifer system are shown in Figure 1. Sampling was carried out during distinct field campaigns representing wet and dry seasonal conditions (the wet season coinciding with the southern hemisphere summer, December to March, and the dry season with winter, June to September), covering different groundwater-flow environments within the karst system. The sampling design was intended to capture groundwater from distinct hydrogeological settings within the karst system, where naturally exposed cave waters, resurgence zones and operational private wells were used to access the aquifer (Figure 2).

Wet-period samples included BI-02 and IR-03 (both from the resurgence zone of Pratinha cave’s system) and BI-04 (well) and IR-01 (phreatic zone of Lapa Doce II cave— “entrance pond”) from Iraquara town; BI-06 (well), IR-05 (well) and IR-06 (well) from Mulungu do Morro town; and IR-07 (well) from Seabra town. Dry-period samples included BI-23 (phreatic zone of Lapa Doce 2 Cave— “Poço do Lima”; Figure 2A), BI-262 (well) and BI-28 (well) from Iraquara town; BI-27 (well) from Seabra town; and BI-30 (well) from Mulungu do morro town. Detailed information and images of sampling sites are presented in Supplementary Table S1 and Supplementary Figure S2.

Hydrochemical characterization was performed for all groundwater samples. Amplicon sequencing of the 16S rRNA gene was performed on 10 samples that had enough DNA for downstream analyses (BI-02, BI-23, BI-262, BI-27, BI-30, IR-01, IR-03, IR-05, IR-06, and IR-07). Genome-resolved shotgun metagenomic sequencing was performed on three samples (BI-04, BI-06, and BI-28), which were selected based on their DNA quantity and quality for metagenomic library preparation and their representation of contrasting hydrochemical conditions across the aquifer. Although an equal number of metagenomic samples from each hydrological period would have been desirable, additional groundwater samples did not yield sufficient DNA quantities for reliable shotgun sequencing and MAG reconstruction. A detailed sampling design and overview of groundwater sites, indicating approximate seasonal comparisons, spatial comparisons, sampling period and sequencing strategy, is presented in Supplementary Table S1.

#### 2.2.1 Groundwater collection and transportation for molecular analyses

For molecular analyses, 20 L of groundwater from each sampling site was collected in previously decontaminated 20-L high-density polyethylene (HDPE) containers (Nalgon, Brazil), transported from the field sites to the car (ranging between 1 and 2 h), transported immediately to the field laboratory on ice in insulated coolers, and kept refrigerated at 4°C until filtration at the temporary field laboratory. Before filling, each decontaminated container was rinsed three times with groundwater from the respective sampling site to condition the container and minimize carryover from the decontamination procedure. The elapsed time between sample collection and filtration ranged from approximately 1–4 h (already accounting for source-to-car transportation). To minimize contamination during sampling and processing, sterile filtration materials and decontaminated collection containers were used throughout all field and laboratory procedures. Containers were decontaminated between sampling events by sequential rinsing with 2% sodium hypochlorite solution, Milli-Q ultrapure water, and 70% ethanol.

The entire 20-L volume collected from each site was filtered through sterile 0.22 μm Sterivex filter units (MilliporeSigma, Burlington, MA, United States) using a multi-channel peristaltic pump (BP600; Milan Equipamentos Científicos, Brazil) to concentrate microbial biomass. No pre-filtration step was applied because samples consisted of oligotrophic groundwater collected from wells and an internal cave lake, with low expected particulate loads. For each sample, 20 L of water were filtered directly through 0.22 μm Sterivex units to maximize microbial biomass recovery.

Groundwater was pumped directly from the collection containers through the filtration system under low-pressure conditions. Between samples, all tubing and filtration lines were decontaminated using the same cleaning procedure applied to the collection containers. Before each filtration (membrane placement), approximately 1 L of groundwater, intentionally collected in excess of the 20-L sample volume, was flushed through the tubing and discarded to remove residual cleaning solutions and condition the filtration line prior to sample processing. A dedicated set of tubing and filtration components was used throughout each sampling campaign and thoroughly decontaminated between consecutive samples following the protocol described above.

Following filtration, Sterivex™ cartridges were filled with RNAlater™ Stabilization Solution (Thermo Fisher Scientific, United States) and stored at −20°C during transport to the Laboratory of Microbial Ecology (LECOM, Brazil), where DNA extraction and downstream analyses were performed.

To assess potential contamination introduced during field sampling, filtration, preservation, transport, and laboratory processing, field filtration blanks were prepared by filtering sterile water through Sterivex™ cartridges using the same tubing, filtration system, preservation solution, and handling procedures applied to environmental samples. Blank filters were preserved, extracted, and processed alongside groundwater samples throughout the workflow. DNA concentrations in all blank controls remained below the detection limit of the quantification method and therefore were not subjected to downstream sequencing. No amplification was observed in procedural controls, and therefore blank samples were excluded from downstream analyses.

All cave sampling activities were conducted under authorization of the Chico Mendes Institute for Biodiversity Conservation (ICMBio permit no. 76989-1).

### 2.3 Hydrochemical characterization

Measurements of pH, oxidation–reduction potential (ORP), electrical conductivity (EC) and dissolved oxygen (DO) were performed in situ using a multiparameter probe Professional Series (YSI, United States) for all groundwater samples collected during wet and dry seasonal campaigns.

For BI samples, bicarbonate concentrations were determined in the field by acidimetric titration using an automatic titrator (HACH, United States). Concentrations of dissolved Ca^2+^, Mg^2+^, SO_4_^2–^, and H_2_S were determined by Eurofins ASL laboratory (wet season) and by the Controle Analitico laboratory (dry season) both laboratories located in Brazil and using ion chromatography and spectrophotometric methods following standard methods and USEPA protocols. All hydrochemical samples were filtered in the field through a 0.45 μm pore membrane and refrigerated until delivered to the laboratory. Anion samples were collected in 60 mL volumes, while cation and H_2_S analyses were performed with volumes of 200 and 250 mL, respectively. For cation analysis, samples were acidified with HNO_3_, while for H_2_S analysis, zinc acetate and NaOH were added to preserve the samples and prevent oxidation of the measured parameter. Hydrogen sulfide was determined using SMEWW 23rd Ed. Methods 4500-S^2–^ H (Limit of Quantification—LOQ = 0.0020 mg/L; wet season) and 4,500-S^2–^ G (LOQ = 0.1 mg/L, dry season). Due to instrumental instability during analysis, major ion data for the “IR” designated samples provided unreliable results and were excluded from this study.

Hydrochemical data treatment and calculation of derived geochemical parameters were performed using AquaChem and PHREEQC software. Comparative analyses involving ion distributions were conducted using only samples with equivalent analytical datasets.

### 2.4 DNA extraction and sequencing

Groundwater collection, filtration procedures, and contamination-control measures are described in detail in section 2.2.1.

Environmental DNA was extracted using the DNeasy PowerWater kit (Qiagen, Germany) according to the manufacturer’s protocol. DNA concentration was quantified using a Qubit fluorometer with the dsDNA High Sensitivity Assay Kit (Thermo Fisher Scientific, United States), and samples meeting the minimum DNA requirements were selected for sequencing (Supplementary Table S2). Procedural controls, including filtration membrane blanks and laboratory bench controls, were processed alongside samples. DNA concentrations in control samples were below the detection limit of the Qubit dsDNA High Sensitivity assay, indicating no detectable background DNA contamination during sample processing.

For amplicon-based community analyses, the V4–V5 region of the 16S rRNA gene was amplified using the primer pair 515F/926R (Caporaso et al., 2011; Walters et al., 2015; Parada et al., 2016). Amplicon libraries were sequenced in two independent Illumina MiSeq (Illumina, San Diego, CA, United States) runs. Samples from the IR series were sequenced using paired-end 2 × 150 bp, while BI-series samples were sequenced using paired-end 2 × 250 bp, generating an average sequencing depth of approximately 100,000 paired-end reads per sample.

For shotgun metagenomic sequencing, libraries were prepared using the NextSeq DNA Library Prep Kit (Illumina, San Diego, CA, United States) and sequenced on the Illumina NextSeq platform with paired-end reads (2 × 100 bp), yielding approximately 80 million paired reads per sample.

Raw metagenomic and 16S rRNA gene amplicon sequencing reads were deposited in the NCBI Sequence Read Archive (SRA) under BioProject accession number PRJNA1463332.

### 2.5 16 S rRNA gene data processing and community analysis

Initial processing of raw paired-end 16S rRNA gene sequences (samples IR-01, IR-03, IR-05, IR-06, IR-07, BI-02, BI-23, BI-262, BI-27, and BI-30) was performed in QIIME 2 (version 2022.2). Sequence quality assessment, filtering, denoising, and amplicon sequence variant (ASV) inference were carried out using the DADA2 plugin implemented in QIIME 2 (Callahan et al., 2016; Bolyen et al., 2019). Forward and reverse reads were truncated to 257 and 251 bp, respectively, and primer sequences were removed by trimming the first 19 bp (forward reads) and 20 bp (reverse reads). Reads were quality-filtered using the default DADA2 expected error threshold (maxEE = 3 for both forward and reverse reads) and denoised using the core DADA2 algorithm. After denoising, forward and reverse reads were merged based on overlap and consistency with the DADA2 error model. Chimeric sequences were then identified and removed using the default consensus method.

The resulting high-quality amplicon sequence variants (ASVs) were used to construct the final feature table, which was used for downstream analyses. Taxonomic assignment was performed against the SILVA database (v138) (Quast et al., 2013) using a naïve Bayes classifier. Although samples were sequenced in independent runs with different read lengths, all datasets were processed using the same bioinformatic workflow and quality-filtering criteria. Subsequent ecological analyses were performed in R (version 3.6.3) using the phyloseq package (McMurdie and Holmes, 2013).

Alpha diversity was assessed using the Shannon diversity index and the Chao1 richness estimator (Chao and Jost, 2012; McMurdie and Holmes, 2014). Data normality was evaluated using the Shapiro–Wilk test. Depending on the distribution of each diversity metric, differences between hydrological periods were assessed using either Student’s *t*-test or the Wilcoxon rank-sum test.

Patterns of beta diversity were explored using principal coordinates analysis (PCoA) based on Bray–Curtis dissimilarity (Paliy and Shankar, 2016). ASV counts were normalized using DESeq2 size factors (sfType = “poscounts”) prior to Bray–Curtis distance calculation. Differences in community composition between hydrological periods were assessed using permutational multivariate analysis of variance (PERMANOVA) implemented in the adonis2 function of the vegan package (Oksanen et al., 2020), with 999 permutations. The PERMANOVA model evaluated hydrological period (wet versus dry) as the explanatory variable.

Homogeneity of multivariate dispersion was assessed using the betadisper function, followed by a permutation test (permutest; 999 permutations) implemented in the vegan package to determine whether differences in community composition were associated with differences in within-group dispersion.

To explore potential relationships between microbial community composition and hydrochemical variables, distance-based redundancy analysis (dbRDA) was performed using the capscale function in the vegan package based on Bray–Curtis dissimilarities. Environmental variables (pH, electrical conductivity, oxidation–reduction potential, and dissolved oxygen) were standardized using z-score scaling prior to analysis. Statistical significance of the constrained model was assessed by permutation tests (999 permutations) using anova.cca. Variance inflation factors (VIFs) were calculated to evaluate multicollinearity among explanatory variables.

To evaluate potential multicollinearity among explanatory variables, variance inflation factors (VIF) were calculated using the vif.cca function. While some variables exhibited moderate collinearity, all were retained in the full model to facilitate exploratory interpretation rather than model reduction, given the limited sample size.

Taxonomic composition was explored using relative abundance profiles generated in phyloseq. ASV tables were converted to relative abundances using the transform_sample_counts function. Taxonomic assignments were summarized at the family level, and taxa lacking family-level annotation were standardized as “Unclassified.” Relative abundances were subsequently aggregated across samples and hydrological periods to facilitate comparisons of dominant community members. Taxonomic composition was visualized using bar plots generated in ggplot2.

ASVs were compared between wet- and dry-period samples based on presence/absence after denoising and quality filtering. Shared ASVs were defined as sequence variants detected in at least one sample from both hydrological periods, whereas exclusive ASVs were detected only within a single period. Relative abundance distributions of shared and exclusive ASVs were calculated from the merged feature table generated by QIIME2.

### 2.6 Metagenomic assembly and genome reconstruction

Raw shotgun metagenomic reads were quality filtered prior to downstream analyses and assembled separately for each of the representative groundwater samples selected for metagenomic sequencing (BI-04, BI-06, and BI-28) using MEGAHIT v1.2.9 (Li et al., 2015). Co-assembly was not performed because the samples represented environmentally distinct groundwater habitats characterized by marked hydrochemical and microbial differences. Independent assemblies were therefore used to maximize recovery of sample-specific genomic information and minimize potential assembly artifacts associated with highly heterogeneous communities.

Only contigs ≥ 1,000 bp were retained for further analyses. Reads from each sample were mapped back to their respective assemblies using Bowtie2 (Langmead and Salzberg, 2012), and resulting SAM files were converted and processed into BAM format using SAMtools (Danecek et al., 2021). The anvi’o (v7) pipeline was used for downstream analyses (Eren et al., 2015). Contig databases were generated with anvi-gen-contigs-database, and open reading frames were predicted using Prodigal (Hyatt et al., 2010).

Coverage profiles were generated with anvi-profile, and individual profiles were merged when appropriate. Taxonomic signals based on single-copy core genes (SCGs) were incorporated into the contigs database to support downstream binning and refinement. Genome binning was initially performed using CONCOCT v1.1.0 (Alneberg et al., 2014). Resulting bins were manually refined in anvi’o (anvi-refine) based on differential coverage, GC content, and taxonomic consistency. Multiple rounds of bin curation were conducted, with genome quality (completeness and contamination) evaluated iteratively using CheckM (v1) (Parks et al., 2015) and CheckM2 (Chklovski et al., 2023).

Metagenome-assembled genomes (MAGs) were classified into quality tiers according to the MIMAG standards (Bowers et al., 2017) as high-quality (>90% completeness, < 5% contamination), medium-quality (≥50% completeness, ≤ 10% contamination), or low-quality draft genomes. Only medium- and high-quality MAGs (≥50% completeness and ≤ 10% contamination) were retained for downstream analyses. To assess potential genome redundancy, the 226 retained MAGs were dereplicated using dRep v3.6.2 (Olm et al., 2017). Dereplication was performed using the same quality thresholds adopted throughout this study (−comp 50, −con 10) to maintain consistency with the MIMAG-based genome selection. Primary genome clustering was performed using Mash at 90% ANI (−pa 0.90), followed by secondary clustering using FastANI at a 95% ANI threshold (−sa 0.95), corresponding to the commonly accepted species-level boundary. Genome quality estimates generated with CheckM2 were supplied through the –genomeInfo option to guide representative genome selection, and analyses were parallelized using 40 CPU threads (−p 40). Taxonomic classification of MAGs was performed using GTDB-Tk v2.7.1 (Chaumeil et al., 2020). Metabolic potential was assessed in anvi’o using the anvi-estimate-metabolism workflow. Taxonomic assignments of MAGs followed the GTDB-Tk classification.

MAGs were deposited in Figshare under DOI: 10.6084/m9.figshare.32209623.

### 2.7 Functional annotation and metabolic reconstruction of metagenome-assembled genomes

Functional annotation and metabolic reconstruction of MAGs were based on KEGG ortholog (KO) assignments. Metabolic potential was inferred using KEGG module completeness, focusing on pathways relevant to the environment. All functional analyses were restricted to high- and medium-quality MAGs, as described in section 2.7.

Metabolic reconstruction emphasized pathways associated with the sulfur cycle. Dissimilatory sulfate reduction (DSR) was assessed using the KEGG M00596 module, and thiosulfate oxidation via the SOX system was assessed using the M00595 module. Module completeness was used as an indicator of the presence of pathways in each MAG. MAGs were considered to co-encode sulfate reduction and sulfur oxidation pathways only when both modules (M00596 and M00595) showed 100% completeness in the same genome. This conservative threshold was adopted to minimize false positives arising from incomplete genome recovery or annotation gaps, which are particularly common in Candidate Phyla Radiation (CPR)-affiliated lineages. The metabolic potential was visualized as a bubble plot using the ggplot2 package in R, displaying the average module completeness and the number of MAGs per taxonomic class in samples BI-04, BI-06, and BI-28.

For sulfur-cycle analyses, a conservative 100% KEGG module completeness threshold was used to classify a module as genomically complete. This criterion was selected to minimize false-positive pathway assignments arising from partial gene sets. However, module absence was not interpreted as definitive evidence of metabolic absence, because incomplete MAGs and genome fragmentation may result in false negatives.

### 2.8 Comparative functional profiling of CPR genomes

To further investigate the ecological roles of the dominant groundwater microorganisms, a comparative analysis of genome-encoded metabolic potential was performed using the KEGG module annotations generated by the anvi’o metabolism workflow (v8). The analysis focused on the four most abundant phyla recovered from the genome-resolved metagenomic dataset, comprising Patescibacteriota (CPR), Pseudomonadota, Nitrospirota, and Desulfobacterota.

The metabolism output generated by anvi’o was imported into R (version 4.3.2) using the readr package and integrated with the taxonomic classifications assigned by GTDB-Tk through a MAG-level relational database generated with functions from the dplyr, purrr, stringr, and tidyr packages of the tidyverse ecosystem. Each genome was linked to all annotated KEGG modules together with their corresponding module completeness estimates (stepwise and pathwise completeness), module category, and taxonomic classification.

For each phylum, the frequency of complete KEGG modules was calculated as the percentage of MAGs encoding a complete module according to the stepwise completeness criterion implemented in anvi’o. Mean module completeness values were also calculated across genomes within each phylum. Modules present in at least 90% of MAGs were classified as core metabolic modules, whereas those detected in ≤ 10% of genomes were considered rare or absent, allowing the identification of conserved and reduced metabolic functions across phylogenetic groups.

MAGs were further grouped into two functional categories: Patescibacteriota (CPR) and the metabolically versatile phyla Pseudomonadota, Nitrospirota and Desulfobacterota, which include the majority of sulfur-cycling MAGs recovered in this study. For every KEGG module, the proportion of genomes encoding complete pathways was calculated independently for each group. Differences in module frequencies between groups were used to identify metabolic pathways preferentially associated with sulfur-cycling microorganisms or consistently reduced in CPR genomes.

Individual KEGG modules were further classified into broader functional categories based on KEGG module descriptions, including central carbon metabolism, respiration, sulfur metabolism, nitrogen metabolism, amino acid biosynthesis, vitamin biosynthesis, nucleotide metabolism, lipid metabolism, transport systems, motility, and DNA repair. Functional profiles were summarized as the proportion of complete modules within each category for both CPR and sulfur-cycling lineages.

A heatmap comparing the complete metabolic functions relative frequency in CPR (Patescibacteriota) and sulfur-cycling lineages (Pseudomonadota, Nitrospirota and Desulfobacterota) was made based on KEGG module annotations generated by the anvi’o metabolism workflow.

## 3 Results

### 3.1 Hydrochemical variability across samples

The detailed hydrochemical data are presented in Table 1. Major-ion data were available for the BI samples, whereas these measurements were unavailable for the IR samples because of instrumental instability during the original analyses (Table 1). Among samples with available major-ion data, groundwater hydrochemistry was consistently dominated by calcium bicarbonate- and calcium sulfate-enriched waters, with bicarbonate concentrations ranging from 82.80 to 317.78 mg/L. Calcium concentrations ranged from 15.64 to 423.85 mg/L, while magnesium concentrations varied between 12.27 and 155.50 mg/L. Samples BI-30, BI-06, BI-262, and BI-28 registered the highest ion concentrations and electrical conductivity values.

**TABLE 1:** Hydrochemical characteristics of groundwater samples from the Irecê Basin karst aquifer system.

Sample ID	Season	pH	EC [μ S/cm]	ORP [mV]	DO (%)	Ca [mg/L]	Mg [mg/L]	SO_4_ [mg/L]	H_2_S [mg/L]	HCO_3_ [mg/L]
BI-23	Dry	7.62	558.00	35.50	44.80	56.97	22.26	15.21	< 0.1	225.78
BI-262	Dry	7.01	1,598.00	−233.60	7.60	137.10	80.53	275.99	11.80	286.73
BI-27	Dry	6.87	269.40	−89.10	23.40	15.64	12.27	2.91	< 0.1	82.80
BI-28	Dry	7.35	1,596.00	−101.90	23.70	125.20	51.70	212.87	25.50	203.93
BI-30	Dry	6.81	3,013.00	52.60	34.30	423.85	155.50	1,406.67	< 0.1	317.78
BI-02	Wet	7.63	513.00	81.60	58.20	53.13	19.98	14.50	< 0.0020	222.33
BI-04	Wet	7.39	1,476.00	63.80	29.90	143.56	48.72	262.75	< 0.0020	215.81
BI-06	Wet	6.89	2,500.00	23.10	2.10	351.15	117.06	1,183.22	< 0.0020	271.40
IR-01	Wet	7.37	597.00	42.00	37.80	−	−	−	−	−
IR-03	Wet	7.41	559.00	53.40	65.40	−	−	−	−	−
IR-05	Wet	6.60	2,864.00	−225.50	5.40	−	−	−	−	−
IR-06	Wet	6.70	3,166.00	−112.90	22.30	−	−	−	−	−
IR-07	Wet	7.12	335.00	22.60	62.90	−	−	−	−	−

The table summarizes physicochemical and hydrochemical parameters measured during wet- and dry-season sampling campaigns, including pH, electrical conductivity (EC), oxidation–reduction potential (ORP), dissolved oxygen (DO), bicarbonate (HCO_3_^–^), calcium (Ca^2 +^), magnesium (Mg^2 +^), sulfate (SO_4_^2−^), and dissolved sulfide (H_2_S). Hydrogen sulfide was determined using SMEWW 23rd Ed. Methods 4,500-S^2−^ H [Limit of Quantification (LOQ) = 0.0020 mg/L; wet season] and 4500-S^2−^ G (LOQ = 0.1 mg/L, dry season) (American Public Health Association [APHA] et al., 2017). Due to instrumental instability during analysis, major ion data for the “IR” designated samples provided unreliable results and were excluded from this study.

Sulfate concentrations also exhibited pronounced variation, ranging from 2.91 mg/L in BI-27 to 1,406.67 mg/L in BI-30. Detectable dissolved sulfide (H_2_S) was restricted to dry-period samples BI-262 and BI-28, with concentrations of 11.80 and 25.50 mg/L, respectively, while all remaining samples with available H_2_S presented concentrations below detection limits.

Groundwater pH values were circumneutral to slightly alkaline, ranging from 6.60 to 7.63. Dissolved oxygen varied markedly among samples, from 2.10% in BI-06 to 65.40% in IR-03. Oxidation–reduction potential (ORP) values ranged from -233.60 to 81.60 mV, indicating substantial variability in redox conditions across the sampled sites and electrical conductivity values varying from 269 to 3,166 μS/cm.

Potential differences between hydrological periods were also observed in redox-sensitive parameters (Supplementary Figure S3). Dry-period samples generally showed lower ORP values and higher H_2_S concentrations, while wet-period samples tended to present more oxidizing conditions and low or undetectable sulfide concentrations.

### 3.2 Microbial community composition based on 16S rRNA amplicon sequencing

A total of 10 samples were analyzed using 16S rRNA gene amplicon sequencing. Raw sequencing depth ranged from 62,772 to 134,272 reads per sample, of which 10,213 to 58,575 reads were retained after quality filtering, corresponding to retention rates between 15 and 44%. Retention rates did not differ significantly between sequencing runs (Wilcoxon rank-sum test, *p* = 0.055), suggesting that read processing affected both datasets comparably despite differences in read length. Despite these reductions, all samples retained more than 10,000 high-quality reads after processing, which was sufficient to recover the majority of detectable diversity as indicated by rarefaction analyses. Rarefaction curves approached asymptotic richness for all samples (Supplementary Figure S4), indicating that sequencing depth was sufficient to recover most of the detectable microbial diversity despite differences in sequencing read length and post-processing read retention. Across all samples, amplicon sequence variant (ASV) richness varied from 90 to 874 ASVs per sample, indicating substantial variability in observed microbial richness among groundwater samples. Detailed information on sequencing output and data recovery across amplicon datasets can be found in Supplementary Table S2.

Alpha diversity metrics did not differ significantly between hydrological periods (*p* > 0.05). Shannon diversity showed a normal distribution (Shapiro–Wilk, *W* = 0.928, *P* = 0.433), whereas Chao1 richness did not meet the assumption of normality (Shapiro–Wilk, *W* = 0.838, *P* = 0.042). Accordingly, Shannon diversity was compared using Student’s *t*-test (*t* = 0.33, df = 8, *P* = 0.74), whereas Chao1 richness was compared using the Wilcoxon rank-sum test (*W* = 9, *P* = 0.53). Although wet-period samples tended to exhibit higher richness and diversity values, these differences were not statistically significant (Supplementary Figure S5 and Supplementary Table S3).

**FIGURE 3:**
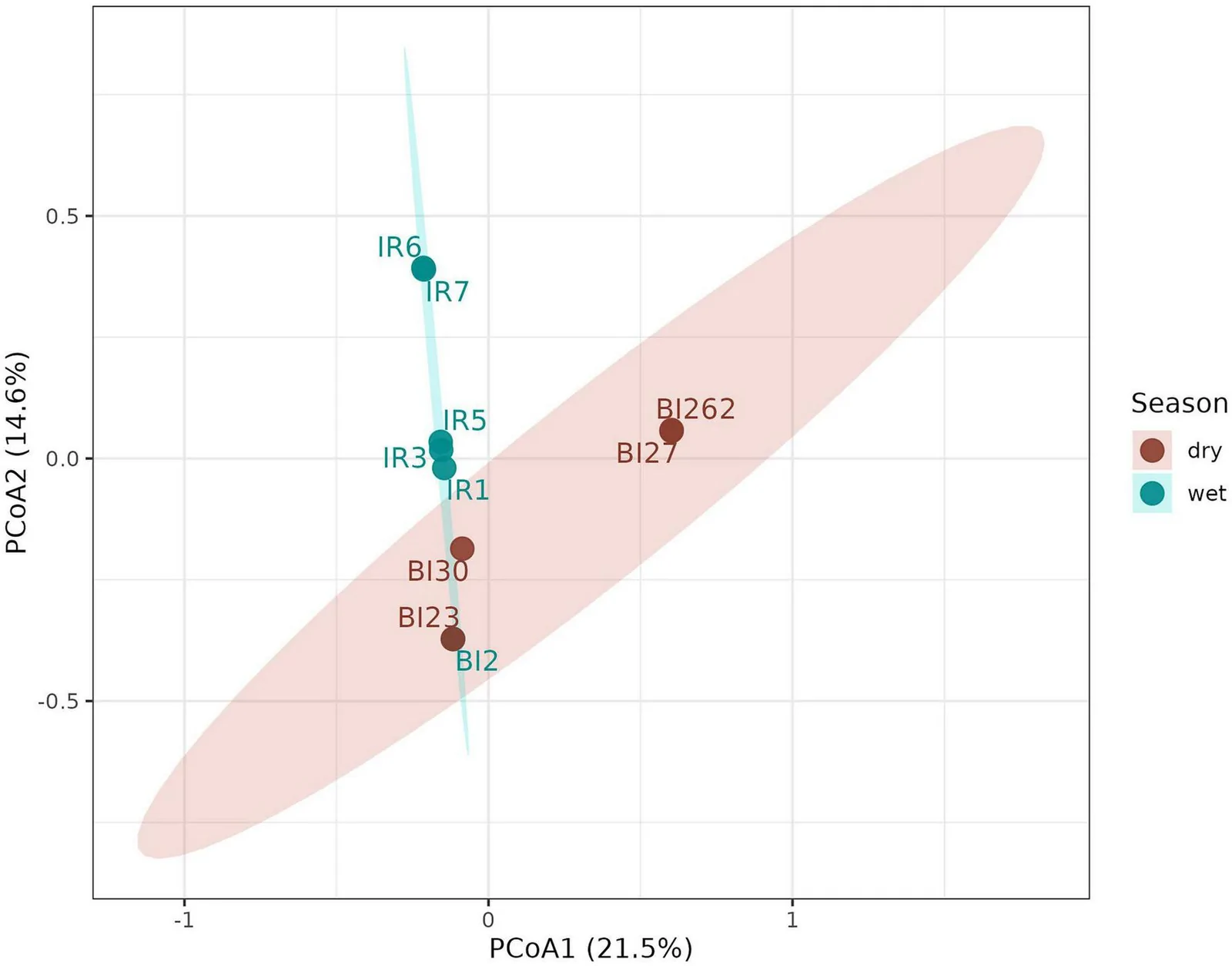
Principal coordinates analysis (PCoA) of 16S rRNA gene amplicon sequence variant composition across groundwater samples using Bray-Curtis distance. Samples are colored according to sampling season (teal for wet and terracotta for dry season). Clustering patterns indicate differences in microbial community composition associated with hydrochemical and seasonal variability (PERMANOVA, *F* = 1.55, *R*^2^ = 0.162, *p* = 0.017).

Principal coordinates analysis (PCoA) based on Bray–Curtis dissimilarities showed that the first two axes accounted for 36.1% of the total variation in the dataset, with the first and second axes explaining 21.5 and 14.6% of the variation, respectively (Figure 3). Community composition differed significantly between samples collected during contrasting hydrological periods (PERMANOVA, *F* = 1.55, *R*^2^ = 0.162, *p* = 0.017), with season explaining approximately 16.2% of the variation in Bray–Curtis dissimilarities. When year and sampling station were included in a full model, the overall effect was not significant (PERMANOVA, *R*^2^ = 0.673, *p* = 0.34), indicating that the observed community variation cannot be attributed to hydrological period alone. Although samples showed partial separation between seasons along the primary ordination axis, there was considerable overlap between groups, consistent with the moderate effect size observed in the PERMANOVA. Multivariate dispersion did not differ significantly between hydrological periods (PERMDISP, *p* = 0.64), indicating that the observed differences in community composition were not driven by differences in within-group variability (Supplementary Figure S6).

Distance-based redundancy analysis (dbRDA) was used to evaluate the relationship between community composition and environmental variables (pH, electrical conductivity, oxidation–reduction potential, and dissolved oxygen) (Supplementary Figure S7). Although the constrained model explained 26.1% of the total Bray–Curtis variation, it was not statistically significant (anova.cca, *F* = 1.065, *p* = 0.312). The first two constrained axes (CAP1 = 0.6995; CAP2 = 0.4201) showed that the explained variation was spread across two main environmental gradients, but moderate collinearity among variables (VIF = 2.63–5.77) limited the interpretation of individual predictors. Therefore, dbRDA results should be interpreted as exploratory rather than confirmatory evidence of environmental structuring.

**FIGURE 4:**
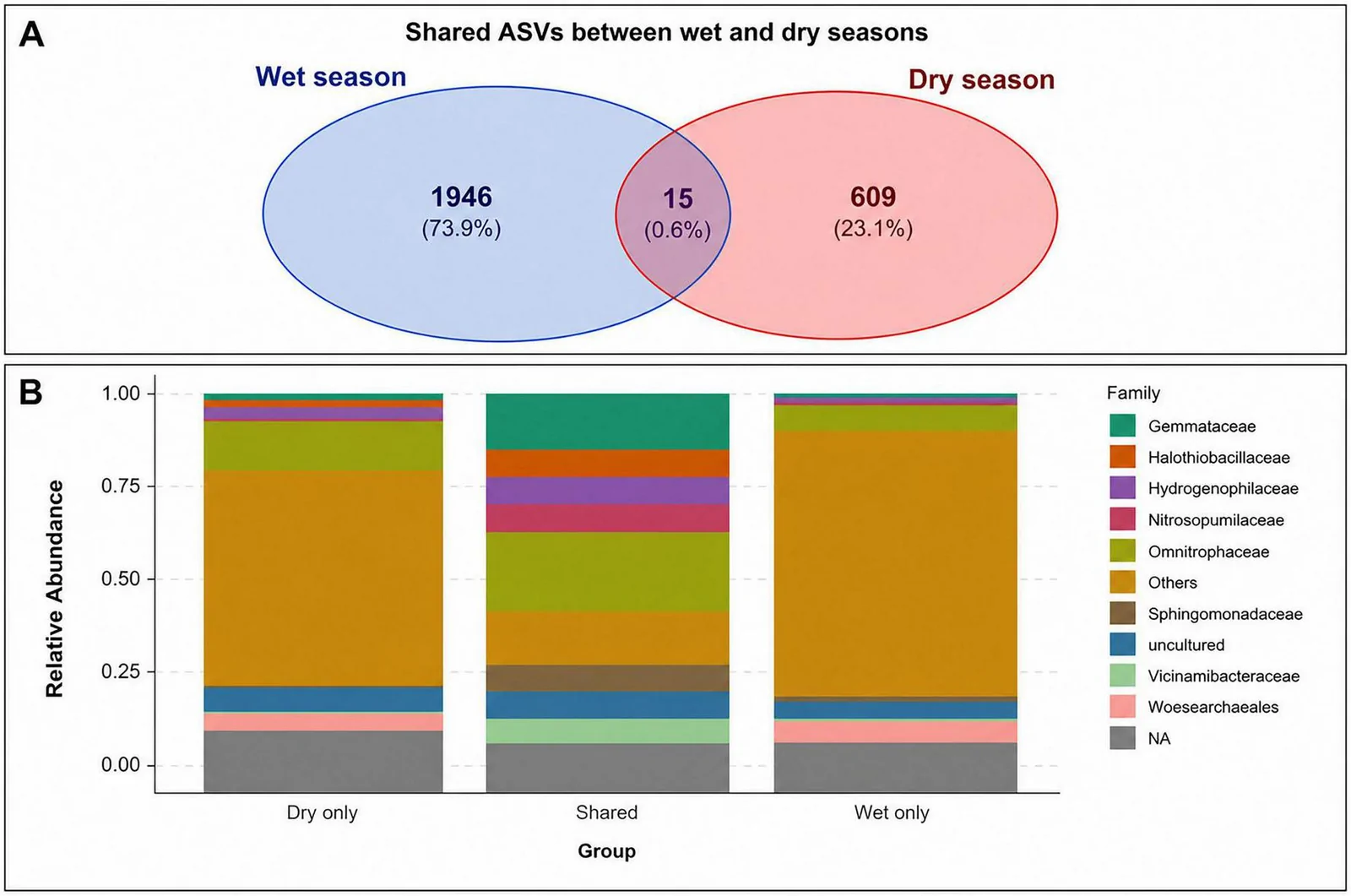
Shared and unique amplicon sequence variants (ASVs) between wet- and dry-season groundwater samples and their taxonomic composition. **(A)** Venn diagram showing the number and proportion of ASVs unique to each season (red for dry and blue for wet seasons) or shared between both conditions. **(B)** Stacked bar plot showing the relative abundance of microbial families associated with dry-season-exclusive, shared, and wet-season-exclusive ASVs. Shared and exclusive ASVs are based on presence/absence across all samples from each season.

Considering the potential influence of differences in sequencing strategy between the IR and BI datasets (section 3.5), we further explored community turnover using ASV presence–absence patterns. It showed that a total of 1,946 ASVs were detected exclusively during the wet season (six samples), while 609 ASVs were unique to the dry season (four samples), and only 15 ASVs were shared between both conditions (Figure 4A). The limited overlap between hydrological periods may reflect differences among groundwater environments, sequencing-related effects, or a combination of these factors.

The taxonomic composition of exclusive and shared ASVs also differed between hydrological periods (Figure 4B). ASVs detected exclusively during the wet period showed greater representation of Proteobacteria, Verrucomicrobiota, and Patescibacteria, whereas Omnitrophaceae, Woesearchaeales, and Parcubacteria were among the most represented groups across both periods. The 15 shared ASVs were distributed among a small number of families, including Omnitrophaceae (3 ASVs) and Gemmataceae (2 ASVs). Vicinamibacteraceae, Halothiobacillaceae, Pirellulaceae, Hydrogenophilaceae, Nitrosopumilaceae, and Sphingomonadaceae were represented by one shared ASV each, while three ASVs could not be classified at the family level and were assigned only to higher taxonomic ranks.

**FIGURE 5:**
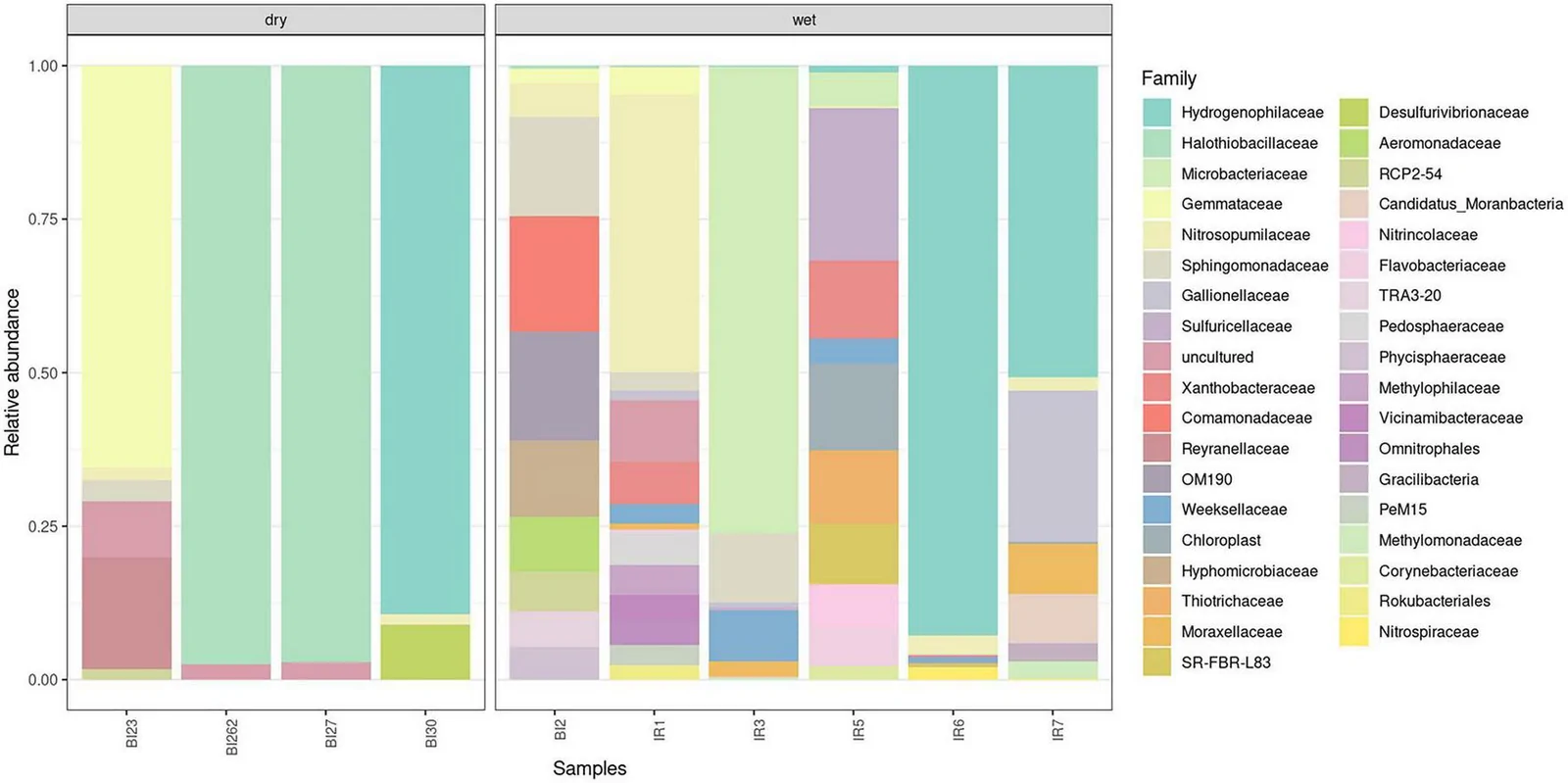
Relative abundance of 16S rRNA gene amplicon sequence variants at the family level across groundwater samples. Stacked bar plots show the relative abundance of microbial families in individual samples grouped according to seasons (wet and dry). Colors represent distinct microbial families based on 16S rRNA amplicon taxonomic assignments.

Relative abundance patterns further revealed substantial differences among individual groundwater samples (Figure 5). Wet-period samples generally showed more evenly distributed relative abundances, whereas dry-period samples tended to exhibit stronger dominance by a limited number of families. In BI-02, no single family dominated the community, with Comamonadaceae (6.7%), OM190 (5.8%), Sphingomonadaceae (4.7%), Omnitrophaceae (2.9%), Vicinamibacteraceae (2.9%), and Gemmataceae (2.8%) contributing at relatively similar proportions. In contrast, BI-23 was mainly composed of Gemmataceae (35.3%), Omnitrophaceae (12.3%), and Reyranellaceae (10.0%), while BI-262 was strongly dominated by Halothiobacillaceae (90.6%), with all remaining families individually representing < 3% of the community.

Considerable variability was also observed among samples collected within the same hydrological period. BI-23, BI-262, BI-27, and BI-30 exhibited distinct taxonomic profiles despite being sampled during the dry period, with Halothiobacillaceae strongly dominating both BI-262 and BI-27, whereas Hydrogenophilaceae predominated in BI-30 and Gemmataceae and Reyranellaceae in BI-23. Wet-period samples likewise differed markedly in their dominant taxonomic groups, with Comamonadaceae and OM190 prominent in BI-02, Nitrosopumilaceae in IR-01, Microbacteriaceae in IR-03, Sulfuricellaceae in IR-05, and Hydrogenophilaceae in IR-06 and IR-07 (Figure 5). These within-period differences highlight substantial spatial and habitat-level heterogeneity among groundwater environments.

### 3.3 Genome-resolved microbial diversity

Three samples (BI-04, BI-06, and BI-28) were subjected to shotgun metagenomic sequencing, yielding between 84.5 and 114.7 million raw paired-end reads per sample. After quality filtering, 96–97% of reads were retained, corresponding to 81.6–110.3 million high-quality reads per sample. Metagenomic assembly and binning resulted in the recovery of 36–97 medium- and high-quality metagenome-assembled genomes (MAGs) per sample. Detailed information on sequencing output, data recovery and sequencing depth is provided in Supplementary Table S2.

Only medium- and high-quality MAGs (≥ 50% completeness and ≤ 10% contamination) were retained for downstream analyses (Supplementary Table S4), as described in Materials and methods (section 3.6). A total of 226 MAGs were recovered across all samples, including 97 from BI-04, 36 from BI-06, and 93 from BI-28. To evaluate genome redundancy, the dataset was dereplicated at 95% average nucleotide identity (ANI) using dRep, resulting in 185 representative MAGs, including 77 from BI-04, 34 from BI-06, and 74 from BI-28, corresponding to an overall reduction of 18.1% in genome number. This result indicates relatively limited redundancy among the reconstructed genomes recovered from the three groundwater metagenomes.

**FIGURE 6:**
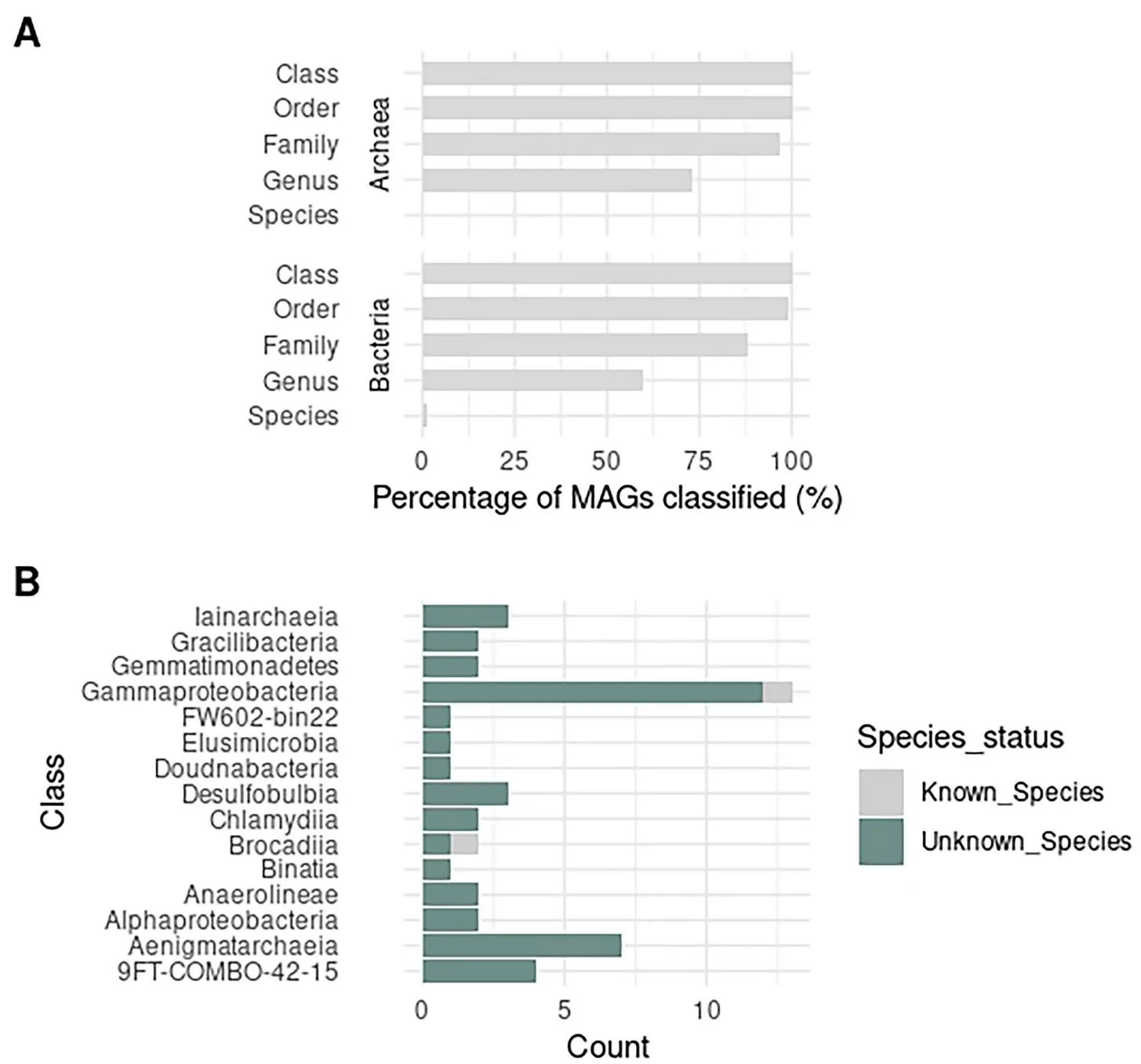
Taxonomic resolution and novelty of metagenome-assembled genomes (MAGs) recovered from the groundwater samples. **(A)** Proportion of bacterial and archaeal MAGs classified at different taxonomic ranks based on GTDB-Tk assignments. Classification success was high up to the family level but decreased markedly at the genus and species levels. **(B)** Distribution of MAGs across taxonomic classes according to species-level assignment status. Bars represent MAGs assigned to known species (gray) or lacking species-level classification (green), highlighting the high taxonomic novelty of the recovered genomes. Taxonomic assignments follow GTDB-Tk classification.

The dereplicated dataset remained predominantly bacterial (83.8%), whereas archaeal MAGs represented 16.2% of the recovered genomes. Taxonomic assignments were highly resolved at upper hierarchical levels, with all MAGs classified at the class rank and nearly all assigned to order (99.4% of bacterial and 100% of archaeal MAGs) and family (88.4 and 96.7%, respectively) (Figure 6A). Taxonomic resolution declined toward lower ranks, with genus-level assignments obtained for 60.0% of bacterial and 73.3% of archaeal MAGs. Species-level classification was rare, with only 1.1% of dereplicated MAGs assigned to previously described species, highlighting the large proportion of undescribed microbial diversity recovered from the aquifer.

Approximately 51.9% of the dereplicated MAGs belonged to candidate or poorly characterized microbial lineages, predominantly members of Patescibacteriota, which represented the most abundant phylum recovered from the aquifer (91 MAGs), dominating the recovered community in BI-04 (50 MAGs) and BI-28 (33 MAGs), while remaining abundant in BI-06. Other recurrent groups included Nanobdellota, Micrarchaeota, Nitrospirota, Pseudomonadota (predominantly Gammaproteobacteria), and Desulfobacterota (Supplementary Table S5).

Community composition differed considerably among samples. BI-06 yielded fewer representative genomes overall (34 MAGs; 18.4% of the dereplicated dataset) but included numerous representatives of metabolically relevant groups, particularly Pseudomonadota (11 MAGs) and Desulfobacterota (3 MAGs). In contrast, BI-04 and BI-28 accounted for 41.6 and 40.0% of the dereplicated MAGs, respectively, and were relatively enriched in Candidate Phyla Radiation (CPR) lineages. Patescibacteriota represented 64.9% of the dereplicated genomes recovered from BI-04 (50 of 77 MAGs) and 44.6% of those recovered from BI-28 (33 of 74 MAGs), indicating an important contribution of ultra-small and metabolically reduced microorganisms to these groundwater communities.

Although CPR lineages dominated BI-04 and BI-28, members of Nitrospirota, Desulfobacterota, and Pseudomonadota were recovered from multiple samples, suggesting the persistence of recurrent sulfur- and nitrogen-associated populations across distinct sectors of the aquifer.

### 3.4 Functional potential of MAGs

**FIGURE 7:**
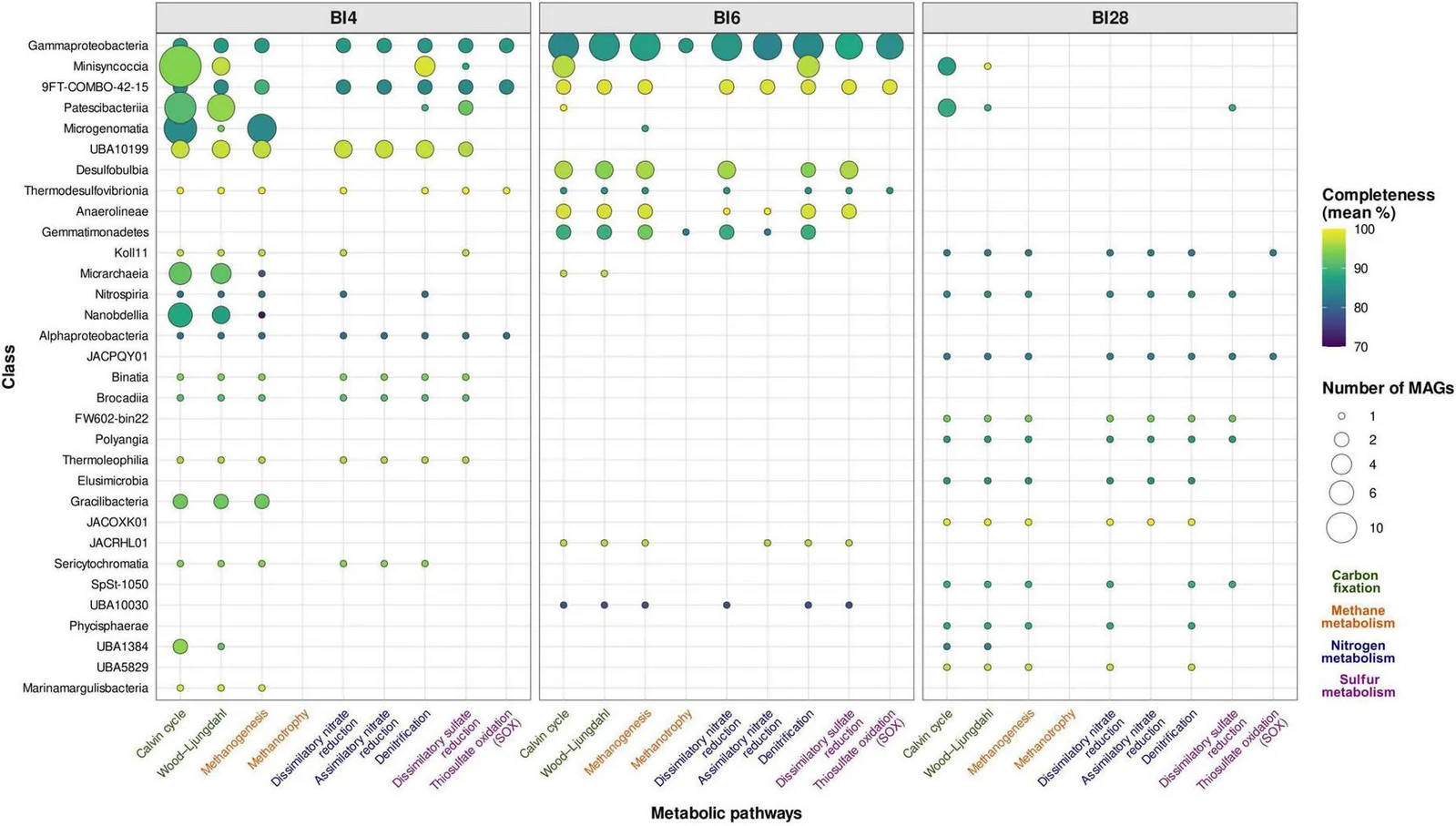
Metabolic potential inferred from metagenome-assembled genomes (MAGs). Distribution of metabolic pathways associated with carbon, nitrogen, and sulfur cycling across 185 medium- and high-quality MAGs based on KEGG module annotation. Bubble size indicates the number of MAGs encoding each pathway, while color represents average genome completeness. Taxonomic assignments were obtained using GTDB-Tk.

Functional potential inferred from KEGG-based annotations revealed a broad but uneven distribution of metabolic pathways across the reconstructed MAGs (Figure 7). Across all samples, core metabolic functions related to central carbon metabolism were detected, including components of the tricarboxylic acid (TCA) cycle, pentose phosphate pathway, and Entner–Doudoroff pathway. However, these pathways were often incomplete at the module level, with missing steps distributed across different MAGs, consistent with incomplete pathway reconstruction across individual genomes, particularly among CPR-associated lineages.

Additional pathways related to cofactor and vitamin biosynthesis, carbohydrate utilization, and basic energy metabolism were variably distributed across samples, with BI-06 showing a comparatively higher representation of these functions despite its lower number of recovered MAGs. In contrast, BI-28 exhibited a more restricted functional repertoire, dominated by incomplete central metabolic pathways and DSR-associated functions.

Comparative analysis across MAGs shows a clear functional contrast. BI-04 and BI-06 genomes have more metabolically diverse communities, including both oxidative and reductive sulfur pathways, while BI-28 genomes have a more limited functional profile dominated by incomplete central metabolism and sulfate-reduction-associated genes. Given the central role of sulfur in the hydrochemistry of the aquifer, and as related pathways emerged as a major component of the functional repertoire, these pathways were examined in more detail in the following section.

These observations refer only to the three metagenomic datasets analyzed and therefore should not be interpreted as representing all groundwater communities sampled during each hydrological period.

### 3.5 Sulfur-cycling functional groups

Because genome-resolved metagenomic analyses were restricted to three representative groundwater samples, the sulfur-cycling pathways described below should be interpreted as sample-specific observations reflecting the hydrochemical conditions of BI-04, BI-06, and BI-28 rather than as general functional characteristics of wet- or dry-period groundwater communities across the aquifer.

**FIGURE 8:**
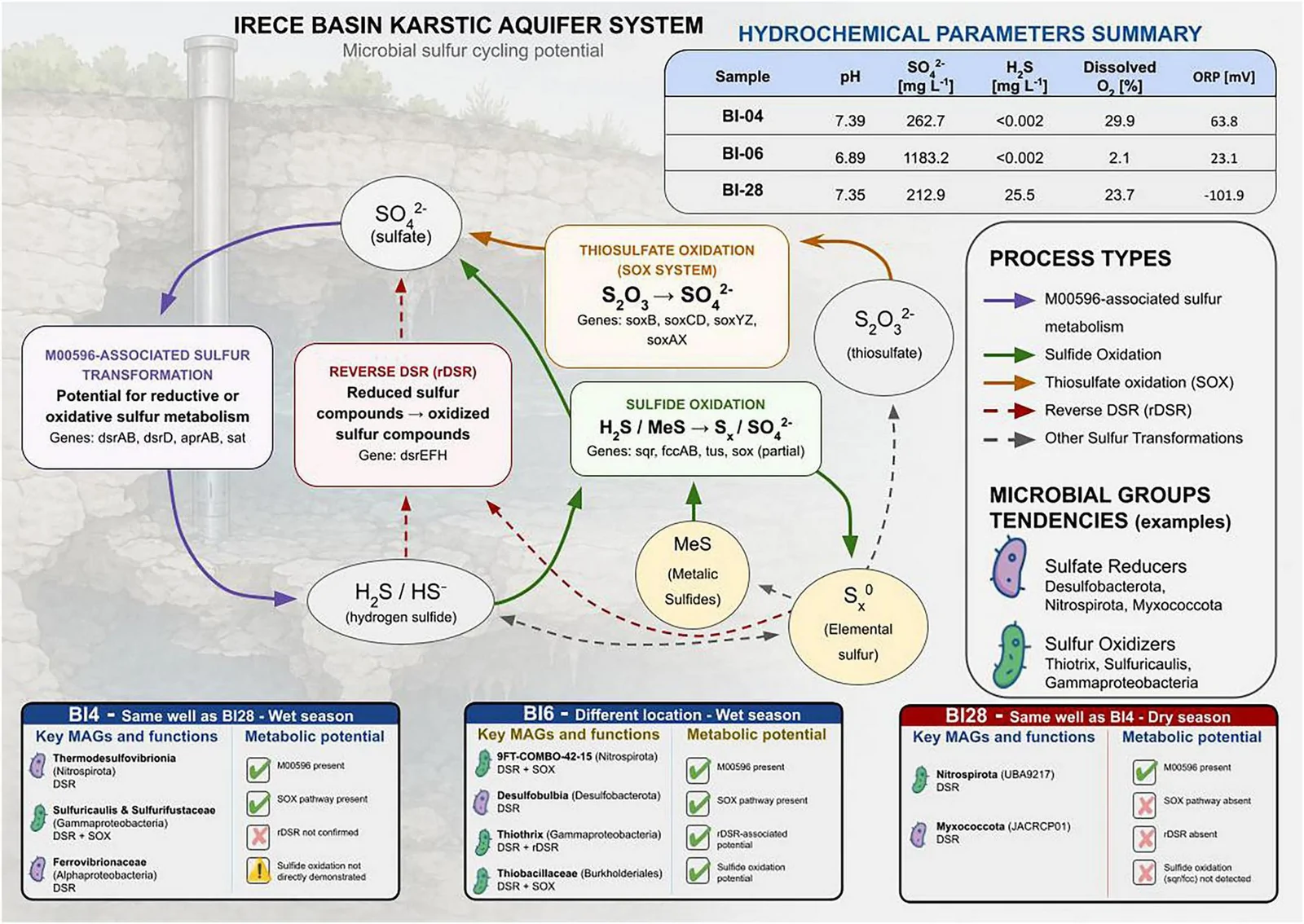
Reconstructed sulfur-cycling potential from the groundwater genomes. The central scheme summarizes sulfur-transformation pathways inferred from MAGs, including M00596-associated sulfur metabolism, thiosulfate oxidation via the SOX system, sulfide oxidation (SQR/FCC), and reverse dissimilatory sulfite reductase (rDSR). Bottom panels compare the distribution of sulfur-cycling metabolic potential among samples BI-04, BI-06, and BI-28. The upper right panel presents a summary of the main hydrochemical parameter variation among the samples. Sulfur oxidation-associated metabolic potential was detected in BI-04 and BI-06 but was absent or poorly represented in BI-28, where dissolved hydrogen sulfide was detected. The background graphical element is purely illustrative and does not contain scientific information.

The distribution of sulfur-related metabolic pathways among MAGs revealed a functional differentiation across the three groundwater samples, consistent with the hydrochemical and redox distinct characteristics described in section 4.1 and summarized in Figure 8.

Three sulfur-metabolism profiles were identified based on the occurrence of the M00596 sulfur-transformation module and the SOX system (M00595): (i) MAGs encoding M00596 without SOX, (ii) MAGs encoding SOX without M00596, and (iii) MAGs encoding both modules simultaneously (Supplementary Table S6). Because DsrAB can participate in either reductive or oxidative sulfur metabolism, the presence and completeness of M00596 alone were not considered sufficient to establish the direction of DsrAB-dependent sulfur transformation.

Conversely, the absence of a complete module was interpreted cautiously, as pathway components may be missing from reconstructed MAGs because of genome incompleteness or assembly fragmentation. Therefore, the functional profiles presented here represent conservative estimates of genomic metabolic potential rather than definitive evidence for the absence of specific sulfur-metabolism pathways.

#### 3.5.1 Genomic potential for dissimilatory sulfur metabolism

The genomic potential for DSR was assessed by the completeness of the KEGG M00596 module, encompassing ATP sulfurylase (*sat*/K00958), APS reductase (*aprAB*/K00394–K00395) and dissimilatory sulfite reductase DsrAB (K11180–K11181). Because DsrAB can operate in either the reductive or oxidative direction, complete M00596 modules were interpreted as evidence for the genomic potential for this sulfur-transformation pathway but not as definitive evidence of sulfate reduction without additional genomic context (Anantharaman et al., 2018).

In BI-04 genomes, four MAGs contained a complete M00596 module. The highest quality MAG was BI-04_MAG_63 (99.82% completeness, 2.25% contamination), associated with Thermodesulfovibrionia (Nitrospirota). The three other MAGs that encode DSR in BI-04 were BI-04_MAG_163 (Pseudomonadota, Alphaproteobacteria; 80.89%), BI-04_MAG_84 (*Sulfuricaulis*, 88.85%) and BI-04_MAG_200 (Sulfurifustaceae, 83.33%), which also encoded the SOX system (see section 3.5.3).

Ten MAGs contained a complete M00596 module, representing the greatest diversity of M00596-associated sulfur-transformation potential among the three samples. Three MAGs associated with Desulfobulbia (Desulfobulbales) were recovered: BI-06_MAG_45 (100% completeness), BI-06_MAG_50 (99.97%), and BI-06_MAG_31 (88.74%). In addition, this sample also contained a MAG from Thermodesulfovibrionia (BI-06_MAG_25; 85.63%), two MAGs from Gammaproteobacteria (BI-06_MAG_102 and BI-06_MAG_109), and four MAGs that had both DSR and SOX modules (BI-06_MAG_32, BI-06_MAG_41, BI-06_MAG_46, and BI-06_MAG_52).

In BI-28 genomes, only two MAGs had the complete DSR module: BI-28_MAG_75 (Myxococcota, class SpSt-1050; 88.92%) and BI-28_MAG_64 (Nitrospirota, class UBA9217; 68.73%). This indicates that complete M00596-associated sulfur-transformation modules were less frequently recovered in BI-28. Overall, no complete SOX modules were detected in this sample, contrasting with samples BI-04 and BI-06. These sulfur-metabolism profiles and their associated taxonomic groups are summarized in Figure 8.

#### 3.5.2 Thiosulfate oxidation via the SOX system

The thiosulfate oxidation pathway (KEGG module M00595), mediated by the SOX multienzyme complex (*soxA*, *soxB*, *soxC*, *soxD*, *soxX*, *soxZ*), was detected exclusively in BI-04 and BI-06 genomes, being absent in MAGs of sample BI-28.

In BI-06, only one MAG encoded the SOX system without DSR: BI-06_MAG_35, linked to the genus *Ideonella* (Gammaproteobacteria, Burkholderiales; 92.45% completeness, 4.19% contamination).

In BI-06, only one MAG encoded the SOX system without a complete M00596 module. The absence of SOX-encoding MAGs in BI-28 coincides with comparatively higher reducing conditions (ORP = −101.9 mV), the presence of dissolved H_2_S (25.5 mg/L), and lower sulfate concentrations compared to the BI-04 and BI-06 samples.

#### 3.5.3 Co-encoding of DSR and SOX modules

Six MAGs, two from BI-04 and four from BI-06, contained complete M00596 and M00595 modules. No MAGs co-encoding DSR and SOX modules were recovered from BI-28.

In sample BI-04, the two MAGs that co-encode the DSR and SOX modules were associated with the Sulfurifustaceae family (Gammaproteobacteria, Acidiferrobacterales): BI-04_MAG_84, assigned to the genus *Sulfuricaulis* (88.85% completeness, 3.35% contamination), and BI-04_MAG_200, assigned to a new lineage at the genus level (SCRL01; 83.33% completeness and 4.16% contamination).

In BI-06, four MAGs co-encoded DSR and SOX modules. Two of them were associated with class 9FT-COMBO-42-15, within the phylum Nitrospirota: BI-06_MAG_32 (99.15% completeness and 0.01% contamination) and BI-06_MAG_41 (97.68% completeness and 1.86% contamination). A third MAG was BI-06_MAG_52, associated with a lineage not classified at the genus level (*PFJX01*) within the order Burkholderiales (Gammaproteobacteria; 88.4% completeness, 1.77% contamination).

The fourth MAG that co-encodes complete M00596 and M00595 modules in BI-06 was BI-06_MAG_46, associated with the genus *Thiothrix* (class Gammaproteobacteria, order Thiotrichales; 98.36% completeness and 0.87% contamination). Members of *Thiothrix* are known to possess both the SOX system and the reverse Dsr (rDsr) complex involved in sulfur oxidation (Ravin et al., 2021). Therefore, in this MAG, the co-occurrence of M00595 and M00596 is more appropriately interpreted as evidence of genomic potential for multiple sulfur-oxidation systems rather than independent reductive and oxidative pathways. However, this interpretation relies on the taxonomic and genomic context of *Thiothrix*, because DsrAB directionality cannot be established from M00596 completeness alone.

The co-encoding of the DSR and SOX modules was limited to BI-04 and BI-06, while BI-28 only had DSR-associated MAG, which had a lower redox potential (ORP = −101.9 mV) and measurable dissolved H_2_S (25.5 mg/L), compared to BI-04 (ORP = 63.8 mV; H_2_S < 0.0020) and BI-06 (ORP = 23.1 mV; H_2_S < 0.0020). The integrated sulfur-cycling potential inferred for each sample is summarized in Figure 8.

### 3.6 Functional differentiation between CPR and sulfur-cycling MAGs

To further investigate the ecological roles of the dominant groundwater microorganisms, KEGG metabolic modules were compared between Patescibacteriota (89 MAGs) and sulfur-cycling lineages represented by Pseudomonadota, Nitrospirota, and Desulfobacterota (28 MAGs). Supplementary Figure S8 shows a comparison between the complete metabolic functions’ relative frequency in CPR (Patescibacteriota) and sulfur-cycling lineages (Pseudomonadota, Nitrospirota and Desulfobacterota). This functional comparison highlighted the pronounced reduction in the metabolic repertoire of CPR genomes relative to sulfur-cycling taxa.

The largest functional differences were associated with respiration, central metabolism and biosynthetic pathways rather than sulfur metabolism itself (Figure 7). Modules related to respiratory metabolism were absent from CPR genomes but were detected in 75.0% of sulfur-cycling MAGs. Likewise, lipid metabolism (0 vs. 34.5%), central carbon metabolism (8.5% vs. 34.9%), vitamin biosynthesis (0 vs. 23.3%), amino acid biosynthesis (4.0% vs. 24.2%), and nucleotide biosynthesis (20.6% vs. 29.9%) were consistently more complete among sulfur-cycling genomes than among CPR genomes. The principal functional differences are summarized in Table 2, highlighting the marked depletion of respiratory and anabolic pathways in CPR genomes.

**TABLE 2:** Comparative functional summary of candidate phyla radiation (CPR) genomes and metabolically versatile groundwater lineages recovered from the Irecê Basin karst aquifer.

Biological function	Representative KEGG modules	CPR genomes (%)	Metabolically versatile MAGs (%)	Ecological implication
Respiration	M00157 (F-type ATPase), M00417 (cytochrome o ubiquinol oxidase)	0	75	Respiratory metabolism is largely absent from CPR genomes, indicating limited autonomous energy conservation.
Central carbon metabolism	M00001 (Glycolysis), M00002 (Glycolytic core), M00009 (TCA cycle)	8.5	34.9	Incomplete central carbon metabolism is consistent with streamlined genomes and reduced metabolic autonomy.
Sulfur metabolism	Sulfate reduction and sulfur oxidation modules	0	11.8	Complete sulfur-cycling KEGG modules were not detected among CPR MAGs and occurred in 11.8% of metabolically versatile MAGs.
Nitrogen metabolism	Nitrate/nitrite transformation modules	0	17.7	Nitrogen transformations are uncommon among CPR genomes.
Amino acid biosynthesis	M00020 (Serine), M00018 (Threonine), M00017 (Methionine), M00016 (Lysine), M00022 (Shikimate pathway)	4	24.2	Extensive reduction of anabolic pathways is consistent with amino acid auxotrophy.
Vitamin/cofactor biosynthesis	M00115 (NAD), M00125 (Riboflavin), M00127 (Thiamine), M00126 (Tetrahydrofolate), M00122 (Cobalamin)	0	23.3	Reduced biosynthetic capacity suggests dependence on metabolites produced by neighboring microorganisms.
Nucleotide biosynthesis	M00048 (Purines), M00049 (AMP/ATP), M00050 (GMP/GTP), M00051 (Pyrimidines), M00053 (dNTP synthesis)	20.6	29.9	Nucleotide metabolism is comparatively more conserved than other anabolic pathways.
Lipid metabolism	M00093 (Phosphatidylethanolamine biosynthesis), M00082–M00083 (Fatty acid biosynthesis)	0	34.5	Reduced membrane lipid biosynthesis further supports extensive metabolic streamlining.

Percentages represent the proportion of genomes within each group encoding complete KEGG metabolic modules according to the anvi’o metabolism workflow. Functional categories summarize the principal metabolic differences identified between CPR (Patescibacteriota) genomes and metabolically versatile lineages (Pseudomonadota, Nitrospirota and Desulfobacterota).

Mining individual KEGG modules identified nine pathways that were nearly absent from CPR genomes (≤ 10%) while occurring in more than 90% of sulfur-cycling MAGs (Supplementary Figure S8). These included respiratory complexes such as cytochrome o ubiquinol oxidase (M00417) and cytochrome c oxidase (M00155), together with pathways involved in proline degradation (M00970) and several specialized metabolic functions. In contrast, CPR genomes retained only a restricted set of conserved metabolic modules, indicating substantially reduced functional repertoires.

## 4 Discussion

### 4.1 Hydrochemistry potentially structures the aquifer microbiome

The combined hydrochemical, amplicon sequencing, and genome-resolved metagenomic datasets provide complementary evidence suggesting that the Irecê Basin karst aquifer system behaves as a highly heterogeneous subsurface system. Marked differences were observed among samples in hydrochemical conditions, microbial community composition, recovered genomes, and metabolic potential. These patterns are consistent with the influence of hydrological variability and redox fluctuations on groundwater microbial communities.

Because genome-resolved metagenomics was performed only for three representative groundwater samples, whereas amplicon sequencing included a broader set of samples, functional inferences derived from MAGs should not be interpreted as representative of all microbial communities characterized by the 16S dataset. Instead, both datasets provide complementary and correlated perspectives on groundwater microbiology under contrasting hydrochemical conditions.

Samples collected during the wet period generally exhibited more oxidizing conditions, detectable sulfate concentrations, and dissolved sulfide levels below detection limits, whereas BI-28 and BI-262, sampled during the dry period, showed lower redox potential, measurable hydrogen sulfide accumulation, and slightly lower sulfate concentrations (Supplementary Figure S3). The marked shift in the SO_4_^2–^/H_2_S ratio between these sampling periods (Table 1), spanning approximately four orders of magnitude, further highlights the strong geochemical contrast between conditions.

Recharge dynamics likely contribute to the hydrochemical and microbiological patterns observed in the aquifer. Following the precipitation events, meteoric water infiltrates the epikarst and fracture networks before reaching the groundwater system and cave environments. During the dry periods, reduced recharge and longer groundwater residence times likely favor the accumulation of dissolved ions, while dissolved oxygen and redox potential may decline due to limited oxygen renewal, continued microbial respiration and water-rock interactions (Barry-Sosa et al., 2024). In contrast, recharge during the wet season introduces more oxygenated waters into the system. Initial recharge pulses may temporarily mobilize dissolved minerals accumulated under stagnant conditions, followed by dilution and progressively more oxidizing conditions as groundwater circulation increases (Palmer and Palmer, 2012; Wang et al., 2020).

Together, these patterns indicate substantial hydrochemical heterogeneity among groundwater environments, providing an environmental context for the microbial differences described below.

### 4.2 Spatial heterogeneity of groundwater microbial communities

Microbial community composition varied substantially among groundwater samples, with differences also observed among samples collected under the same hydrological conditions. PCoA based on Bray–Curtis distances (Figure 3) showed partial separation between wet- and dry-period samples along the first axis, but with substantial overlap among groups. PERMANOVA indicated a moderate compositional difference between hydrological periods, whereas the full model including sampling year and station was not significant, indicating that the observed variation cannot be attributed to hydrological period alone. No significant differences in multivariate dispersion or alpha diversity (Chao1 and Shannon) were detected between hydrological periods (*p* > 0.05; Figure 3; Supplementary Figures S5, S6). Environmental variables explained 26.1% of the variation in dbRDA (Supplementary Figure S7) but were not significant overall, with interpretation limited by collinearity. The position of BI-02 within the ordination (Figure 3) further illustrates that community similarity cannot be attributed exclusively to hydrological periods, reinforcing the contribution of additional environmental and temporal sources of variation. In this framework, compositional differences among samples likely reflect multiple interacting ecological and environmental processes shaping beta diversity patterns (Baselga, 2010).

The sampling design does not represent a true longitudinal seasonal survey. Only BI-04 and BI-28 correspond to approximate wet- and dry-period sampling of the same groundwater location (Supplementary Table S2), whereas the remaining comparisons involve different groundwater environments sampled during different hydrological periods. Therefore, differences between wet- and dry-period samples cannot be attributed exclusively to seasonal succession and are more appropriately interpreted in the context of spatial heterogeneity, hydrochemical variability, and contrasting hydrological conditions.

Considerable taxonomic variability was also observed among samples collected during the same hydrological period, indicating substantial variation among local groundwater environments rather than a consistent association with the hydrological period. This within-period variability was evident across both dry- and wet-period samples, which frequently exhibited contrasting dominant taxa despite being sampled under the same hydrological classification (Figure 5).

At the taxonomic level (Figure 5), the recurrent detection of Pseudomonadota together with Candidate Phyla Radiation (CPR)-associated lineages across samples suggests the coexistence of metabolically versatile microorganisms and poorly characterized subsurface specialists. Members of Pseudomonadota, including Comamonadaceae, are often linked to various carbon utilization pathways and nitrogen transformations like denitrification and ammonification, which matches the relatively even community structure observed in wet-period samples (Wu et al., 2018). The prevalence of Patescibacteria is also consistent with previous studies in karst and groundwater environments, where CPR lineages frequently represent an important fraction of oligotrophic subsurface microbial communities characterized by reduced genomes and limited metabolic capacities (Tian et al., 2020; Chaudhari et al., 2021; Zhong et al., 2023). The dominance of Halothiobacillaceae in BI-262, a well-known sulfur-oxidizing bacteria capable of using reduced sulfur compounds as energy sources (Whaley-Martin et al., 2019), further illustrates the distinct taxonomic profiles observed among groundwater environments. The potential relationship between sulfur-transforming microorganisms and local redox conditions is discussed in section 4.3.3.

The recurrent occurrence of Patescibacteria and Woesearchaeales is consistent with the presence of microorganisms adapted to oligotrophic environments and potentially dependent on interactions with other community members, as suggested by their reduced genomes (Tian et al., 2020; Srinivas et al., 2024). The persistence of some taxa across samples, including Omnitrophaceae, also suggests the existence of stable micro-niches maintained by relatively consistent geochemical conditions (Stegen et al., 2012; Probst et al., 2018; Sharma et al., 2026).

Although only a small number of ASVs were shared between wet- and dry-period datasets, these included taxa potentially involved in nitrogen and sulfur cycling, indicating that key ecological functions may indicate that some microbial groups associated with nitrogen and sulfur cycling persist across sampling periods. The persistence of these functions likely reflects the continuity of underlying geochemical drivers, such as the availability of nitrogen compounds and sulfur species, which remain present across sites even as their concentrations and redox states fluctuate (Hershey and Barton, 2018; Probst et al., 2018).

The spatial patterns observed in this study likely reflect the interplay of hydrological, hydrochemical, and spatial processes operating within karst aquifers, where geochemical conditions strongly constrain energy availability and may contribute to differences in the relative importance of heterotrophic and chemolithotrophic metabolisms, including sulfur transformations (Griebler and Lueders, 2009; Hershey and Barton, 2018).

### 4.3 Environmental controls on microbial metabolic potential

Because genome-resolved metagenomic analyses were restricted to three representative groundwater samples, the functional interpretations presented below refer only to these datasets and should not be interpreted as general functional characteristics of wet- or dry-period groundwater communities across the aquifer. Rather, they illustrate how metabolic potential varies among representative groundwater microenvironments sampled under contrasting hydrochemical and hydrological conditions, providing insight into possible functional differentiation associated with local environmental settings.

#### 4.3.1 Metabolic fragmentation

Recovered high- and medium-quality metagenome-assembled genomes (MAGs) showed a wide but uneven distribution of metabolic pathways (Figure 7), reflecting the expected functional diversity in a geochemically heterogeneous aquifer (Valentin-Alvarado et al., 2024). Spatial and temporal variations in redox conditions and solute availability likely created distinct ecological niches that shape microbial functional organization across groundwater environments. The high proportion of MAGs assigned to unknown genera and species (Figure 6) highlights the taxonomic novelty of the aquifer microbiome, consistent with previous studies that reported large fractions of uncultivated and poorly characterized microbial lineages in subsurface and groundwater ecosystems (Anantharaman et al., 2016; Castelle and Banfield, 2018; Hershey et al., 2018; Tian et al., 2020; Chaudhari et al., 2021; Zhong et al., 2023).

#### 4.3.2 Reduced metabolic repertoires suggest ecological dependency of CPR bacteria

The predominance of Patescibacteriota (CPR), representing nearly half of all recovered medium- and high-quality MAGs (Supplementary Table S5), prompted a comparative functional analysis to better understand their metabolic potential and ecological role in the karst aquifer.

Comparative functional analysis showed that CPR genomes had substantially reduced metabolic repertoires relative to metabolically versatile groundwater lineages (Supplementary Figure S8), with the strongest differences involving energy conservation and biosynthetic functions rather than sulfur metabolism itself. This pattern was also evident at the individual KEGG-module level, where respiratory and biosynthetic modules were consistently more complete among the non-CPR MAGs (Table 2).

This metabolic profile closely agrees with the genomic characteristics originally described for the Candidate Phyla Radiation by Brown et al. (2015), who demonstrated that CPR bacteria possess unusually small genomes, reduced biosynthetic repertoires and simplified metabolic networks compared with most free-living bacteria. Rather than encoding complete central metabolic pathways, many CPR lineages appear to have undergone extensive genome streamlining, losing numerous anabolic functions while retaining only a minimal set of pathways required for basic cellular maintenance (Brown et al., 2015; Castelle and Banfield, 2018). Genome reduction is now recognized as one of the defining evolutionary features of the CPR radiation and has been consistently reported across phylogenetically diverse lineages occupying groundwater, subsurface and sedimentary environments (Brown et al., 2015; Castelle and Banfield, 2018; Tian et al., 2020).

Subsequent comparative genomic studies have considerably expanded this interpretation. Castelle and Banfield (2018) proposed that the widespread loss of biosynthetic pathways across CPR bacteria reflects an evolutionary transition toward metabolically dependent lifestyles, in which essential metabolites are acquired from neighboring microorganisms rather than synthesized de novo. Comparative evolutionary analyses further suggest that progressive gene loss constrains CPR metabolic autonomy while favoring increasingly intimate ecological associations with metabolically versatile microorganisms (Jaffe et al., 2021). Likewise, groundwater-associated CPR populations consistently exhibit incomplete pathways for amino acid, nucleotide and cofactor biosynthesis while retaining genes required for core cellular processes, supporting metabolic interdependence as a widespread ecological strategy among subsurface CPR lineages (He et al., 2021).

The coordinated reduction observed across multiple functional categories in CPR MAGs (including respiration, central carbon metabolism, amino acid biosynthesis, vitamin biosynthesis and lipid metabolism) suggests that populations of this group inhabiting the Irecê karst aquifer are unlikely to sustain fully autonomous metabolisms, consistent with the reduced metabolic autonomy proposed for CPR bacteria inhabiting oligotrophic environments (Tian et al., 2020). Instead, the presence of many CPR genomes alongside metabolically versatile Pseudomonadota, Nitrospirota, and Desulfobacterota, which together have much broader biosynthetic and respiratory capabilities, is consistent with pronounced functional complementarity within the groundwater microbiome. Although this pattern is consistent with potential metabolic dependency, genome-resolved metagenomics alone cannot demonstrate direct ecological interactions, specific metabolite exchanges, syntrophy, or parasitism. Thus, our results support metabolic dependency as a plausible ecological strategy for CPR populations in the Irecê aquifer, rather than demonstrating it directly.

Such a strategy may represent one possible adaptation to oligotrophic karst aquifers, where low nutrient availability and spatially heterogeneous redox conditions impose strong energetic constraints on microbial growth. Under these conditions, genome reduction coupled with metabolic specialization may reduce biosynthetic costs while enabling CPR populations to exploit metabolites continuously released by neighboring microorganisms. Similar ecological interpretations have been proposed for groundwater, deep subsurface and sediment-associated CPR communities, where streamlined genomes are increasingly viewed as adaptive responses to chronic energy limitation and metabolic specialization rather than simply the consequence of genome erosion (Castelle and Banfield, 2018; Tian et al., 2020; He et al., 2021).

Although several CPR lineages have been proposed to establish episymbiotic associations with other bacteria, the genome-resolved data generated here do not allow direct evaluation of physical interactions, restricting our interpretation to functional complementarity inferred from genome content (He et al., 2021).

#### 4.3.3 Sulfur-cycling metabolic potential across redox gradients

Sulfur-cycling potential varied markedly among the three metagenomic datasets (Figure 7 and Supplementary Table S6) and was consistent with their contrasting hydrochemical conditions (Table 1 and Supplementary Figure S3). Complete M00596-associated modules, comprising *sat*, *aprAB*, and *dsrAB*, were detected in all three samples. MAGs encoding complete DSR modules were affiliated with multiple taxonomic groups, including Nitrospirota, Desulfobacterota, and Gammaproteobacteria, consistent with the broad phylogenetic distribution of sulfate-reducing microorganisms in subsurface environments (Muyzer and Stams, 2008). In contrast, complete SOX modules were detected only in BI-04 and BI-06. Because DsrAB can participate in either reductive or oxidative sulfur metabolism, M00596 completeness was interpreted as evidence of genomic potential for this sulfur-transformation pathway rather than as definitive evidence of sulfate reduction alone.

In BI-04 and BI-06, MAGs encoded both M00596-associated and SOX modules, resulting in greater sulfur-metabolic diversity than observed in BI-28. Several MAGs from these samples co-encoded complete M00596 and SOX modules, indicating the potential for multiple sulfur-transformation pathways within the same genomes. In particular, the *Thiothrix*-affiliated BI-06_MAG_46 contains genomic features consistent with the presence of both SOX and reverse-Dsr systems, as previously reported for this genus (Anantharaman et al., 2018; Ravin et al., 2021). However, co-occurrence of these modules does not by itself demonstrate simultaneous or sequential use of the corresponding pathways.

In contrast, BI-28 exhibited a more restricted sulfur-cycling profile, with M00596-associated modules detected in MAGs but no complete SOX modules. This pattern coincided with the more reducing conditions observed in BI-28, including lower ORP, measurable dissolved H_2_S, and lower sulfate concentrations relative to BI-04 and BI-06. Thus, the distribution of sulfur-cycling potential across the three representative groundwater environments is consistent with differences in local redox conditions, with more oxidizing samples supporting a broader representation of oxidative and reductive sulfur-transformation pathways.

A schematic overview of the sulfur-metabolism profiles and potential discussed in this section is available in Figure 8.

Taken together, these observations indicate that sulfur-cycling potential varies with local hydrochemical conditions among the representative groundwater environments analyzed. The presence of taxa linked to sulfur transformation, such as Desulfocapsaceae, even in low amounts, supports the idea of coupled sulfur cycling, where reduced sulfur compounds are continuously produced and reoxidized across microscale redox gradients (Zhou et al., 2025). However, the three metagenomic datasets provide sample-specific evidence and cannot establish aquifer-wide patterns or demonstrate direct coupling between sulfur transformations. Broader inference regarding the distribution and ecological importance of these pathways will require additional genome-resolved datasets across the aquifer.

#### 4.3.4 Possible implications of sulfur cycling for karst processes

The combined hydrochemical, amplicon-based, and genome-resolved metagenomic datasets consistently indicate that sulfur cycling represents an important component of groundwater biogeochemistry in the Salitre Formation aquifer. Elevated sulfate concentrations, dissolved sulfide accumulation under reducing conditions, and widespread detection of sulfur-related metabolic pathways in representative MAGs indicate the widespread genomic potential for sulfur transformations across contrasting hydrochemical settings. Particularly high sulfate concentrations were detected in samples BI-06 (Lagoa Preta farm, wet period) and BI-30 (Lagoa Preta farm, dry period) (> 1,000 mg/L SO_4_^2–^), while dissolved sulfide accumulation was observed under more reducing conditions, especially in BI-28 and BI-262. These observations suggest that sulfur oxidation and reduction are recurrent processes within the aquifer, although their relative importance likely varies among groundwater microenvironments.

The Salitre Formation carbonates contain disseminated sulfide minerals, including pyrite, galena, and sphalerite, as described in section 3.1, which represent important geological sources of sulfur within the aquifer system. Previous hydrochemical investigations in the region have also reported recurrent high sulfur content and reducing groundwater conditions in wells associated with the Irecê Basin aquifer system since the mid-1980s (Guerra, 1986) and later (Auler, 1999; Valle, 2004; Morita, 2023), indicating that sulfur-related geochemical processes may be a persistent characteristic of the system rather than isolated local anomalies.

Because sulfur oxidation has been associated with carbonate dissolution in several sulfidic karst systems (Hill, 1995; Engel et al., 2004; Barton and Northup, 2007; Jones et al., 2015; De Waele et al., 2016; Turrini et al., 2024), previous geological investigations have proposed sulfuric acid speleogenesis (SAS) as a possible mechanism contributing to cave development in parts of the Salitre Formation (Auler, 1999; Auler and Smart, 2003; Valle, 2004; Morita, 2023; Audra et al., 2026). However, the present study was not designed to evaluate sulfuric acid speleogenesis directly. Instead, our results demonstrate the widespread occurrence of sulfur-transforming microorganisms and sulfur-related metabolic potential, indicating that sulfur cycling is an ecologically important process within the groundwater ecosystem.

Whether these microbial processes contribute to carbonate dissolution or speleogenetic evolution remains an open question. Addressing this hypothesis will require dedicated studies integrating sulfur isotope geochemistry, mineralogical characterization of secondary sulfate minerals, microscale geochemical profiling, and direct measurements of sulfur-related microbial activity.

## 5 Concluding remarks

This study highlights the substantial spatial and hydrochemical heterogeneity of microbial communities across the Irecê Basin karst aquifer. Integrated hydrochemical, amplicon, and genome-resolved metagenomic analyses revealed pronounced variation in microbial composition and metabolic potential across groundwater environments, with local hydrochemical and redox conditions potentially contributing to these patterns. Although the sampling design does not allow the independent effects of hydrological period to be resolved, the observed differences among groundwater environments highlight the importance of spatial and habitat-level heterogeneity in structuring the aquifer microbiome.

Recovered MAGs revealed a groundwater microbiome dominated by poorly characterized and taxonomically novel groups. Sulfur-transformation potential was prominent across the metagenomic samples (Figure 8), with M00596-associated sulfur metabolism detected in all three samples and SOX pathways restricted to the comparatively more oxidizing BI-04 and BI-06 environments. These patterns indicate spatially heterogeneous sulfur-cycling potential that is consistent with differences in local redox conditions. Together, these findings support sulfur transformations as an important component of groundwater biogeochemistry in this sulfur-rich karst system.

Beyond sulfur cycling, comparative functional analyses revealed that the remarkable abundance of Candidate Phyla Radiation (CPR) bacteria is associated with extensive reductions in energy-conserving and biosynthetic pathways, consistent with metabolically streamlined lifestyles. The coexistence of these reduced-genome microorganisms with metabolically versatile groundwater lineages is consistent with potential functional complementarity and ecological dependency, particularly under the energetic constraints imposed by oligotrophic karst environments. However, these genomic patterns do not by themselves demonstrate direct metabolic interactions or metabolite exchange.

In summary, these findings suggest that the Irecê Basin groundwater microbiome is characterized by spatially heterogeneous sulfur-transformation potential and the coexistence of metabolically flexible and genome-streamlined microbial groups. These observations provide new insights into the microbial ecology of sulfate-rich tropical karst aquifers and highlight how local hydrochemical and redox heterogeneity may shape sulfur-related metabolic potential and the functional organization of subsurface microbial communities. The widespread occurrence of sulfur-transforming metabolic potential also suggests that these microbial communities may have the potential to feed back on local geochemical conditions, with possible implications for the evolution of sulfur-rich karst environments and associated speleogenetic processes.

## Data Availability

The datasets presented in this study can be found in online repositories. The names of the repository/repositories and accession number(s) can be found in the article/Supplementary material.
