# Supplementary material for: Groundwater microbial community structure under contrasting hydrological conditions in a sulfate-rich karst aquifer
Source: Front Microbiol. 2026 Sep 9;17:1883366. doi: 10.3389/fmicb.2026.1883366 (PMC13597930; doi:10.3389/fmicb.2026.1883366)
Supplement: Supplementary file 1 [file Data_Sheet_1.pdf]

Supplementary Material

1. Supplementary Figures

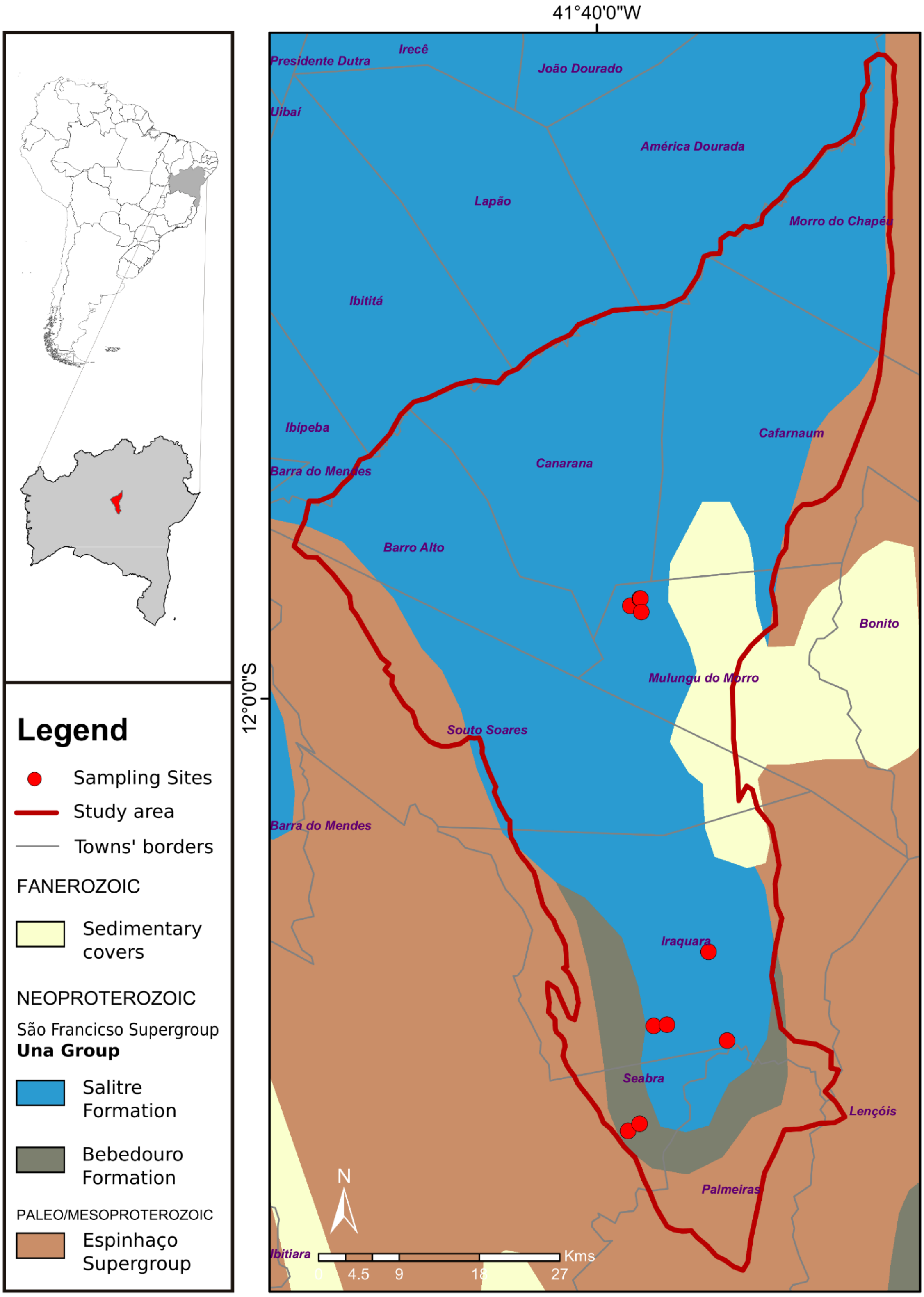

Supplementary Figure S1 – Geological and geographic context of the groundwater sampling sites in the Irecê Basin karst system, Bahia, Brazil. Sampling sites are indicated by red circles within the study area outlined in red. The sampled aquifer is predominantly hosted in carbonate rocks of the Neoproterozoic Salitre Formation (blue), part of the São Francisco Supergroup and the main

karstified unit of the region. Additional geological units include the Bebedouro Formation (gray), siliciclastic rocks of the Espinhaço Supergroup (brown), and localized Phanerozoic sedimentary covers (light beige). Municipal boundaries are indicated by gray lines. Inset maps show the location of the study area within Bahia State and Brazil. Adapted from (GÓMEZ TAPIAS et al., 2019).

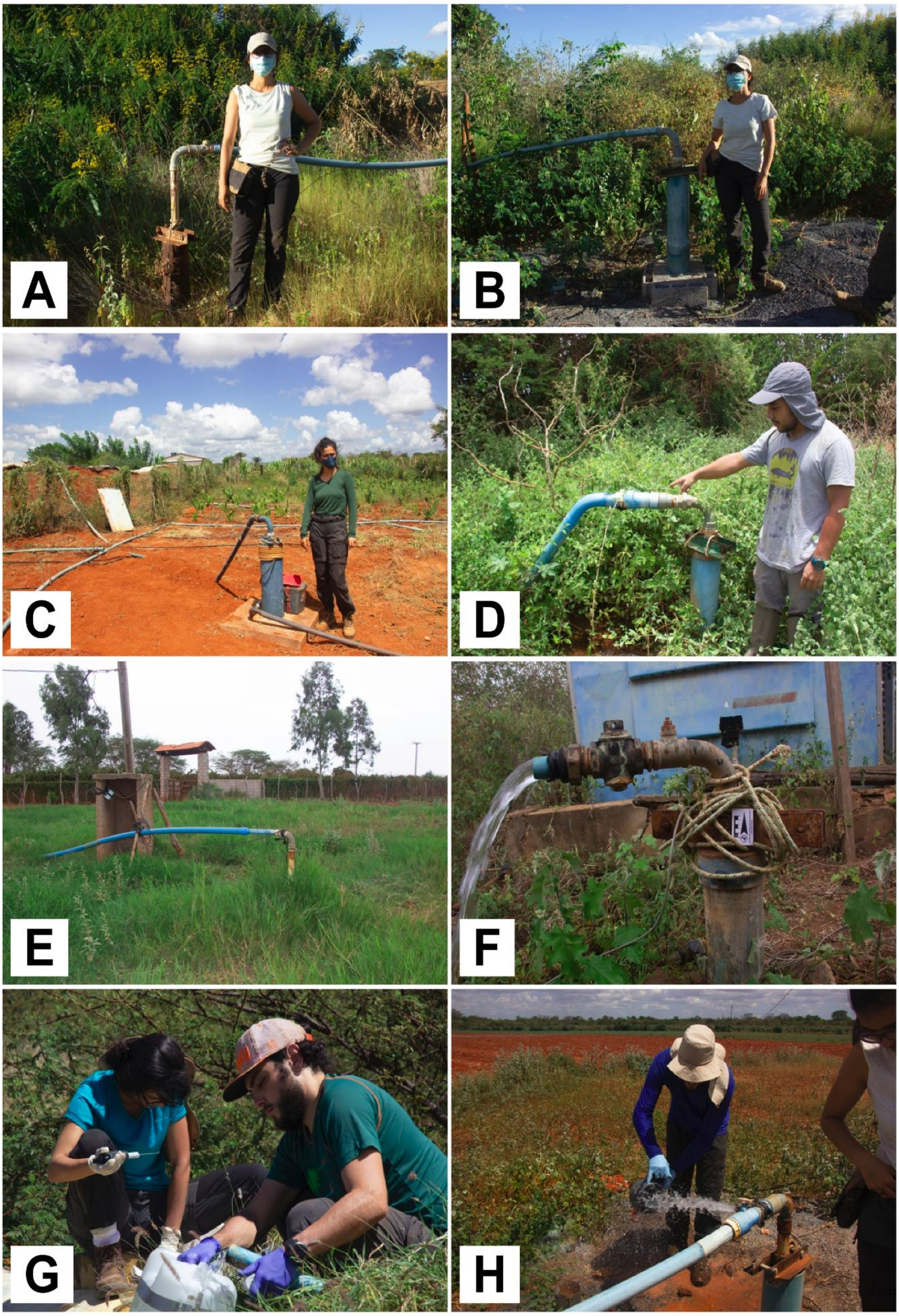

Supplementary Figure S2 – Groundwater sampling sites and hydrogeological settings within the Iraquara karst aquifer system, Bahia, Brazil. Sampling sites represent distinct groundwater access conditions within the Salitre Formation karst system during wet- and dry-season field campaigns. (A) Lagoa Preta well 1 (samples IR-05 and IR-06). (B) Lagoa Preta well 2 (samples BI-06 and BI-30). (C) Luisão well (sample BI-262). (D) Seabra well (samples IR-07 and BI-27). (E) Santíssimo village well (samples BI-04 and BI-28). (F–G) Representative images of groundwater discharge and water collection from operational wells. Coordinates, sampling dates, hydrolochemical conditions, and sequencing approaches are summarized in Supplementary Table S1.

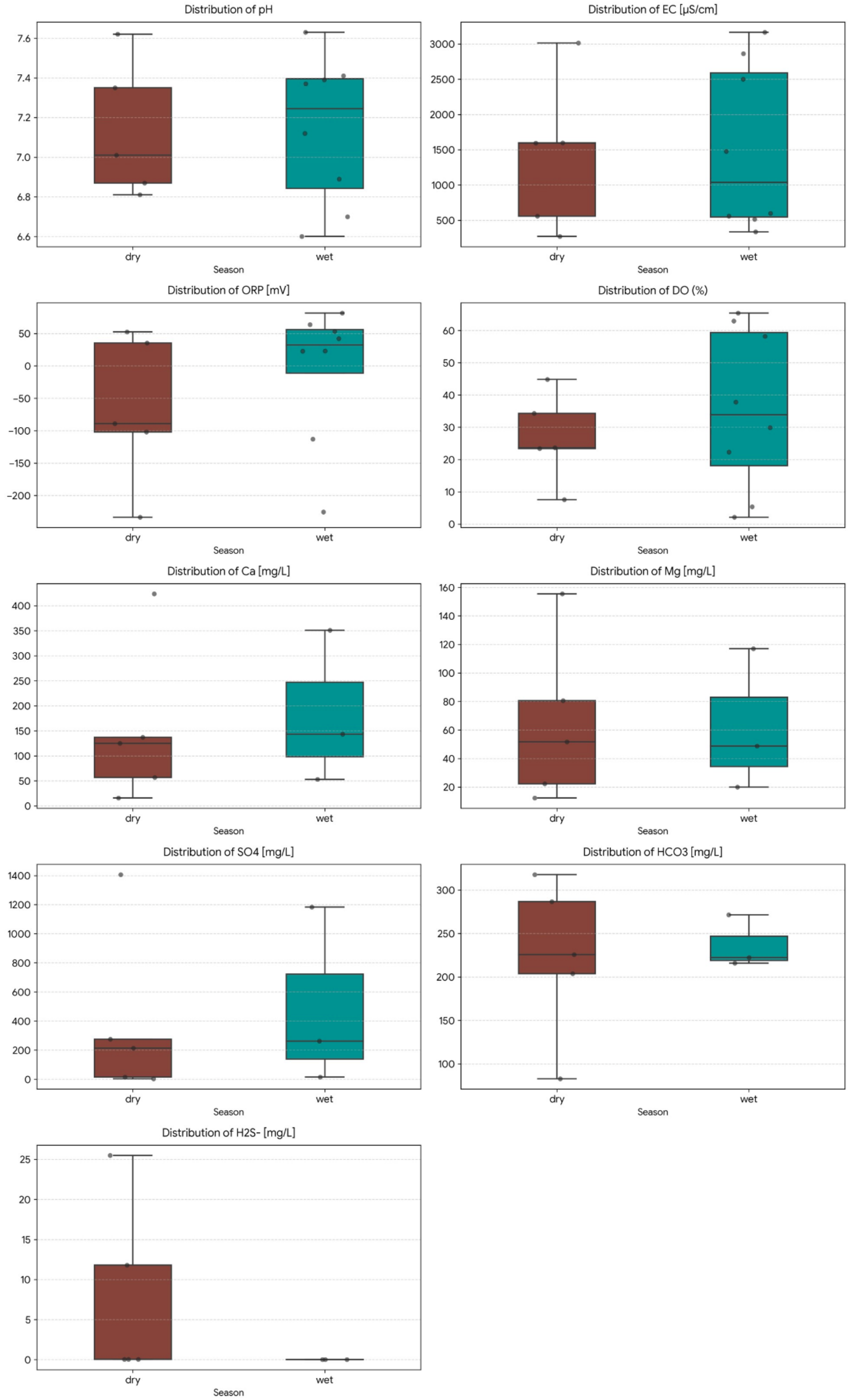

Supplementary Figure S3. Hydrochemical variability across wet- and dry-season groundwater samples. Boxplots show the distribution of pH, electrical conductivity (EC), oxidation–reduction potential (ORP), dissolved oxygen (DO), calcium (Ca^{2+}), magnesium (Mg^{2+}), sulfate (SO_4^{2-}),

bicarbonate (HCO_3^-), and dissolved sulfide (H_2S) in groundwater samples collected during dry and wet seasons. Points represent individual samples. IR samples were not included in the ion distribution boxplots due to differences in analytical datasets between sampling campaigns.

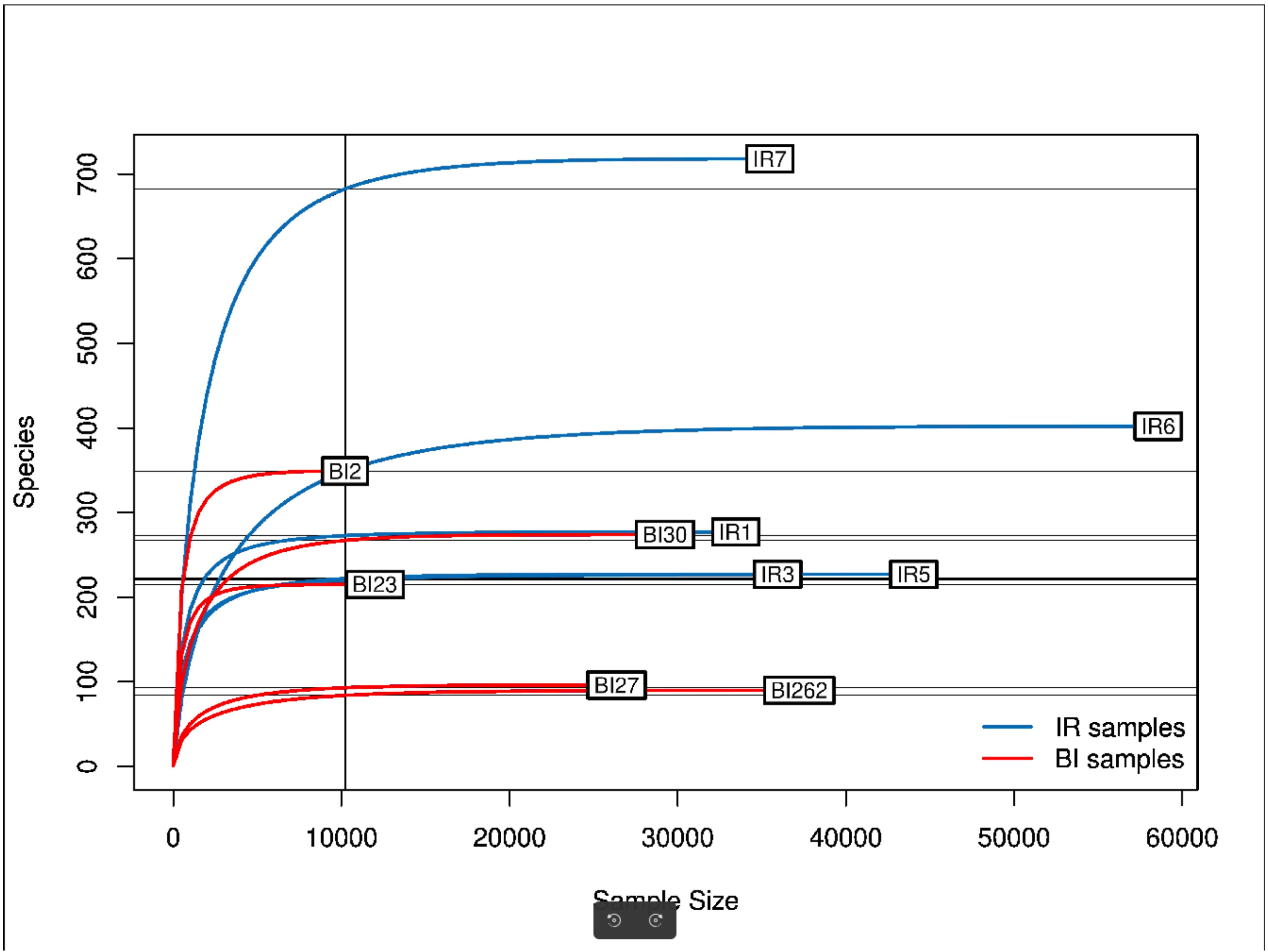

Supplementary Figure S4. Rarefaction curves based on 16S rRNA gene amplicon sequencing data after DADA2 processing. Curves represent the accumulation of amplicon sequence variants (ASVs) as a function of sequencing depth for each groundwater sample. Curves corresponding to the IR series are shown in blue, whereas curves corresponding to the BI series are shown in red. The vertical line indicates the minimum sequencing depth among samples (10,143 reads), corresponding to the rarefaction threshold shared by all samples.

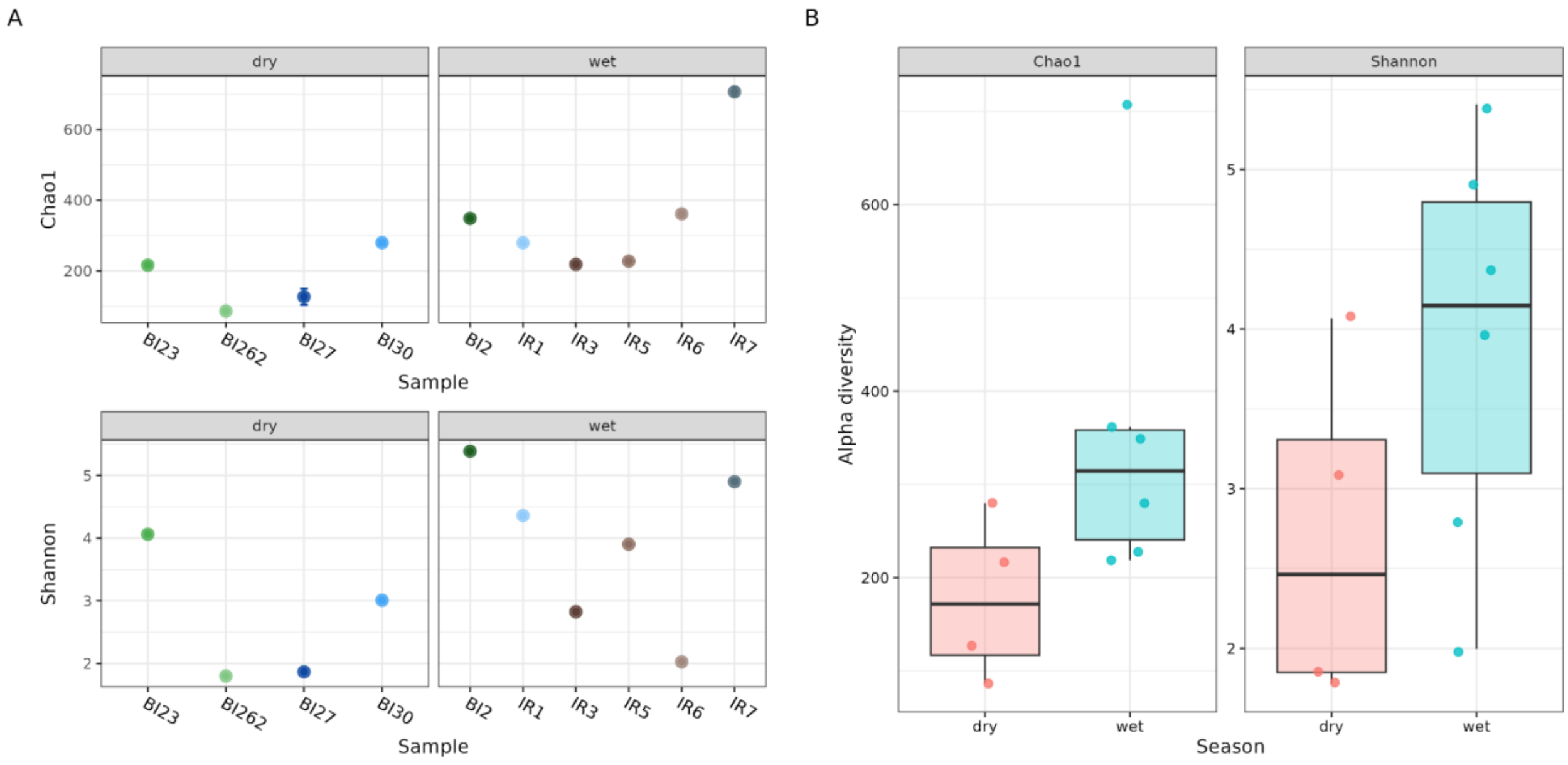

Supplementary Figure S5. Alpha diversity of groundwater microbial communities (16S rRNA gene data) grouped by hydrological period. (A) Chao1 richness and Shannon diversity across individual samples (x-axis: samples; y-axis: diversity estimates). Points represent individual samples. No significant differences were detected between dry and wet periods for either metric (t-test, $p > 0.05$ for Shannon; Wilcoxon rank-sum test, $p > 0.05$ for Chao1). (B) Boxplots showing interquartile ranges and medians; points represent individual samples. No significant differences were observed

between hydrological periods for either diversity metric (Wilcoxon rank-sum test, $p > 0.05$).

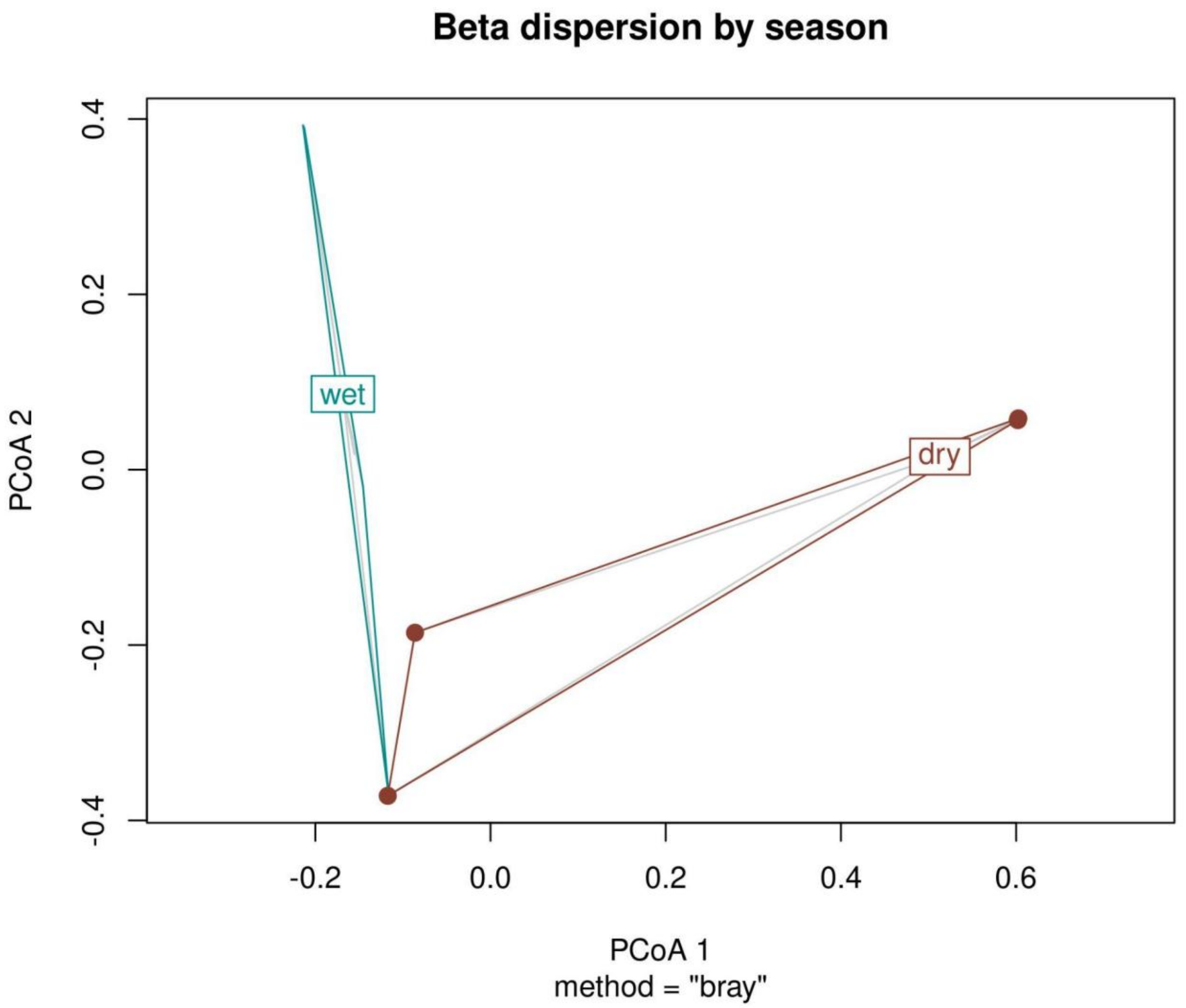

Supplementary Figure S6 – Assessment of multivariate dispersion (PERMDISP) among groundwater microbial communities from wet- and dry-season samples based on Bray–Curtis dissimilarities. Polygons illustrate within-group dispersion used to test the assumption of homogeneity of multivariate dispersion prior to PERMANOVA.

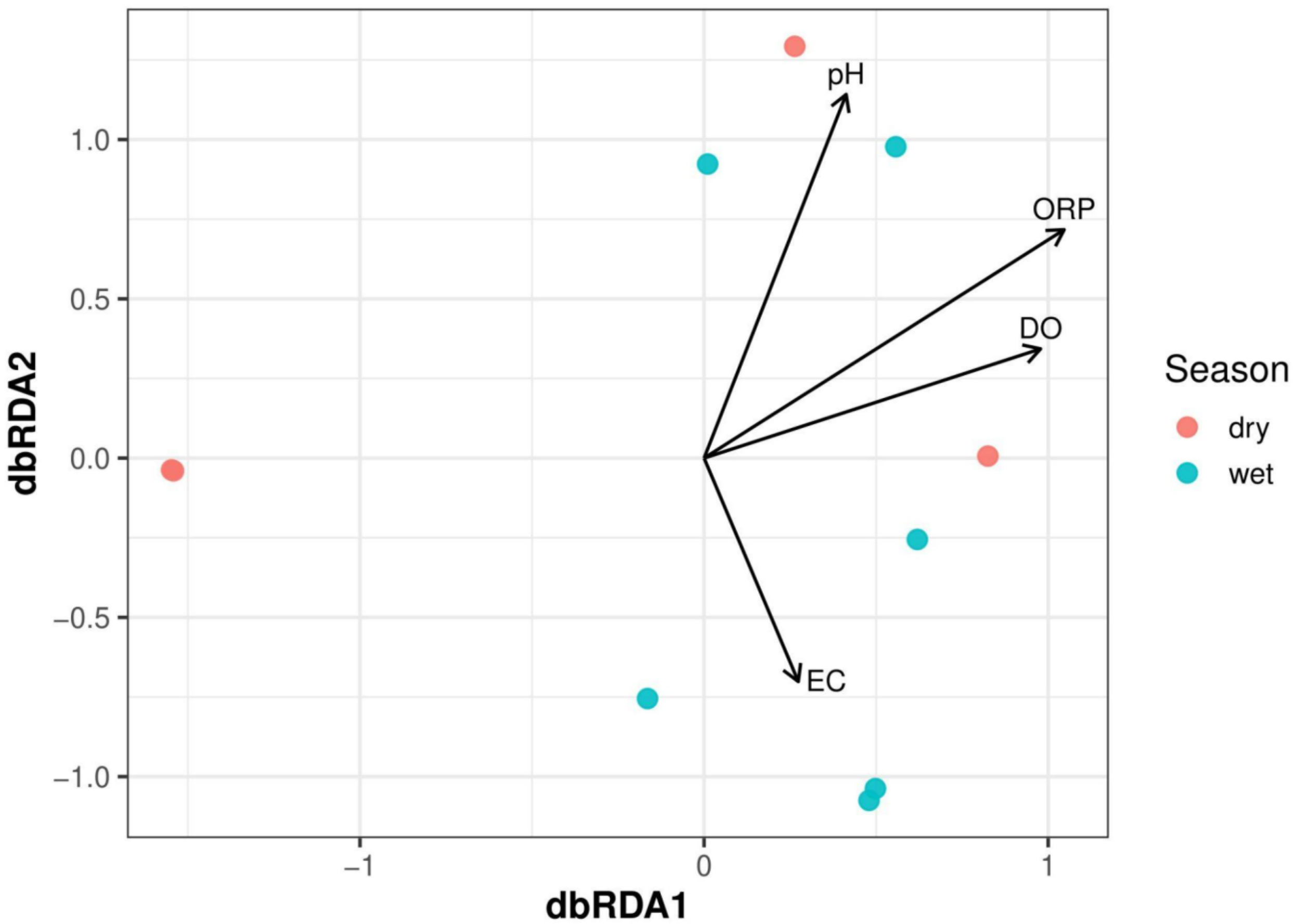

Supplementary Figure S7 – Distance-based redundancy analysis (dbRDA) relating groundwater microbial community composition (16S rRNA gene ASVs) to hydrochemical variables across the sampled groundwater sites. Arrows represent the direction and relative strength of fitted environmental variables, including pH, oxidation-reduction potential (ORP), dissolved oxygen (DO), and electrical conductivity (EC). Points correspond to individual groundwater samples and are colored according to hydrological period (wet and dry). The ordination indicates that variation in microbial community composition is associated with hydrochemical gradients, particularly those related to redox conditions and water chemistry, while also revealing substantial overlap among samples collected during different hydrological periods.

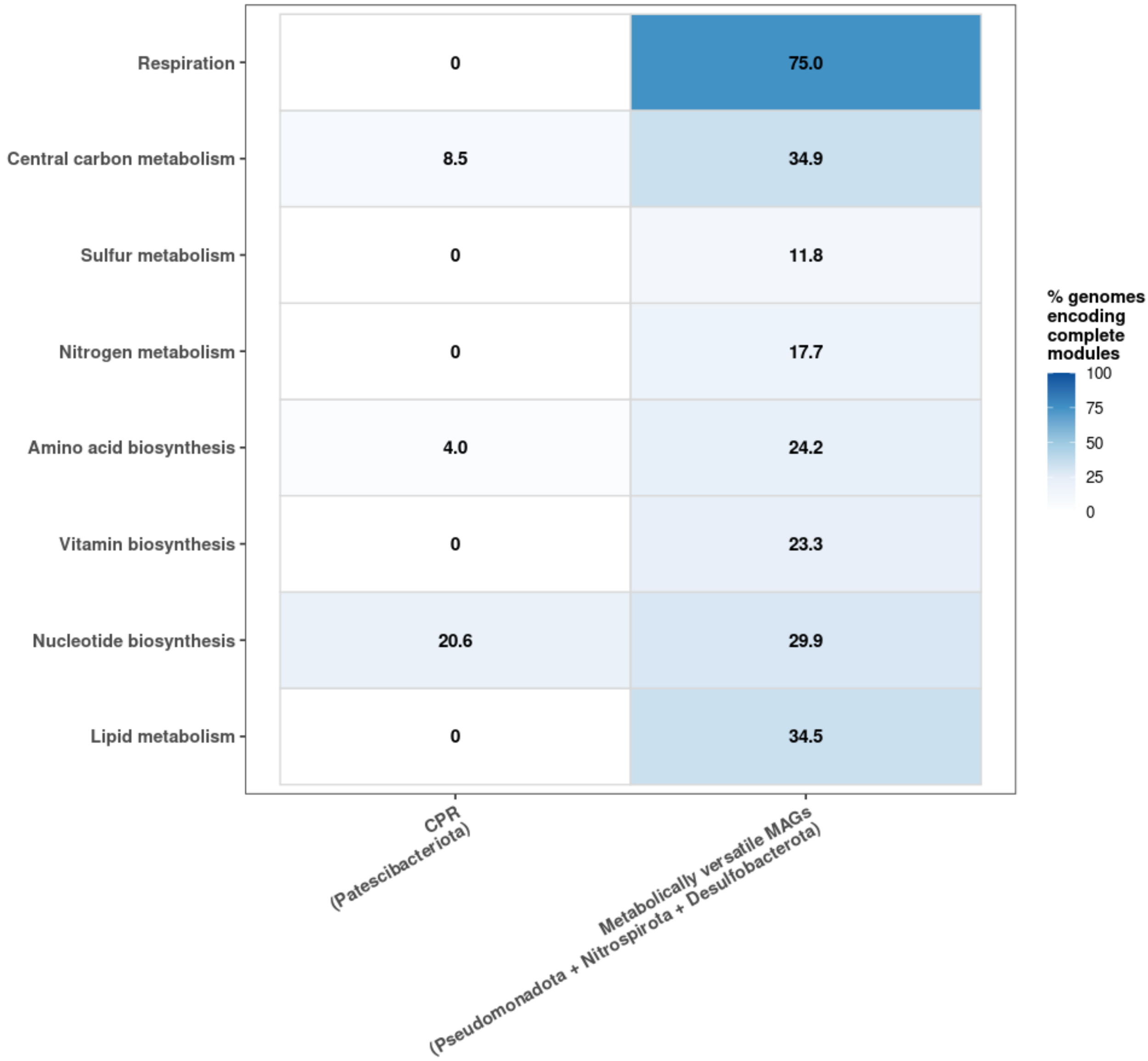

Supplementary Figure S8. Comparative functional profiles of Candidate Phyla Radiation (CPR) genomes and metabolically versatile sulfur-cycling lineages recovered from the Irecê Basin karst aquifer. The heatmap summarizes the relative frequency of complete metabolic functions in CPR (Patescibacteriota) and sulfur-cycling lineages (Pseudomonadota, Nitrospirota and Desulfobacterota). Functional categories were derived from KEGG module annotations generated by the anvi'o metabolism workflow. Values represent the percentage of genomes within each group encoding complete KEGG modules according to stepwise module completeness.

2. Supplementary Tables

Supplementary Table S1 – Sampling design and overview of groundwater sites, indicating approximate seasonal comparisons, spatial comparisons, sampling period and sequencing strategy. Sampling locations, coordinates, collection dates, season, groundwater access type, sequencing strategies and sample series are summarized for all analyzed samples. Spatial correspondence indicates samples collected from the same groundwater system or locality but not necessarily from identical groundwater flow paths or wells. Only BI-04 and BI-28 represent repeated sampling of the same well under contrasting hydrological conditions.

Sample ID	Group	Town	Location	Season	Origin	Sequencing type	Sample series	Sequencing run	Corresponding sample(s)	Comparison category	Latitude	Longitude	Date
IR-01	B	Iraquara	Lapa Doce II cave – entrance pond	Wet	Phreatic zone	16S amplicon	IR	MiSeq 2×150	BI-23	Same cave system (spatial comparison)	-12.33996	-41.61721	Jan/17/2018
IR-03	C	Iraquara	Gruta Azul cave	Wet	Resurgence	16S amplicon	IR	MiSeq 2×150	BI-02	Same resurgence (spatial comparison)	-12.35719	-41.5567	Jan/18/2018
IR-05	D	Mulungu do Morro	Lagoa Preta farm – well 1	Wet	Well	16S amplicon	IR	MiSeq 2×150	BI-06, BI-30	Same hydrogeological sector	-11.91008	-41.65052	Jan/19/2018
IR-06	D	Mulungu do Morro	Lagoa Preta farm – well 1	Wet	Well	16S amplicon	IR	MiSeq 2×150	BI-06, BI-30	Same hydrogeological sector	-11.91016	-41.65123	Jan/19/2018
IR-07	E	Seabra	Cochó do Malheiros village	Wet	Well	16S amplicon	IR	MiSeq 2×150	BI-27	Approximate seasonal comparison (same locality)	-12.44546	-41.64927	Jan/19/2018
BI-02	C	Iraquara	Gruta Azul cave	Wet	Resurgence	16S amplicon	BI	MiSeq 2×250	IR-03	Same resurgence (repeat sampling)	-12.35719	-41.5567	Jan/26/2021
BI-04	H	Iraquara	Santíssimo village	Wet	Well	Shotgun	BI	NextSeq 2×100	BI-28	Direct seasonal comparison (same well)	-12.34187	-41.63055	Jan/27/2021
BI-06	D	Mulungu do Morro	Lagoa Preta farm – well 2	Wet	Well	Shotgun	BI	NextSeq 2×100	IR-05, IR-06, BI-30	Same hydrogeological sector	-11.91016	-41.65141	Jan/29/2021
BI-23	B	Iraquara	Lapa Doce II cave – Poço do Lima	Dry	Phreatic zone	16S amplicon	BI	MiSeq 2×250	IR-01	Same cave system (spatial comparison)	-12.33996	-41.61721	Aug/4/2021
BI-262	F	Iraquara	Quixaba village	Dry	Well	16S amplicon	BI	MiSeq 2×250	None	No corresponding sample	-12.27003	-41.58044	Aug/5/2021
BI-27	E	Seabra	Cochó do Malheiros village	Dry	Well	16S amplicon	BI	MiSeq 2×250	IR-07	Approximate seasonal comparison (same locality)	-12.44991	-41.65659	Aug/5/2021
BI-28	H	Iraquara	Santíssimo village	Dry	Well	Shotgun	BI	NextSeq 2×100	BI-04	Direct seasonal comparison (same well)	-12.34187	-41.63055	Aug/5/2021
BI-30	D	Mulungu do Morro	Lagoa Preta farm – well 2	Dry	Well	16S amplicon	BI	MiSeq 2×250	IR-05, IR-06, BI-06	Same hydrogeological sector	-11.91016	-41.65141	Aug/6/2021

Supplementary Table S2 – Sequencing output and downstream data recovery across amplicon and metagenomic datasets. Raw and quality-filtered read counts, read retention percentages, and downstream outputs are reported for each sample. Amplicon datasets include the number of recovered amplicon sequence variants (ASVs), while metagenomic datasets include the number of medium- and high-quality metagenome-assembled genomes (MAGs) after dereplication.

Sample ID	Sequencing type	Reads (raw)	Reads (filtered)	Retained (%)	ASVs	MAGs
BI-02	16S amplicon	62,772	10,213	16	349	-
BI-04	Shotgun	84,467,456	81,600,700	97	-	77
BI-06	Shotgun	114,711,448	110,274,182	96	-	34
BI-23	16S amplicon	79,448	11,986	15	215	-
BI-262	16S amplicon	92,972	37,215	40	90	-
BI-27	16S amplicon	68,640	26,363	38	96	-
BI-28	Shotgun	96,198,712	92,619,852	96	-	74
BI-30	16S amplicon	86,954	29,235	34	874	-
IR-01	16S amplicon	110,075	33,450	30	277	-
IR-03	16S amplicon	80,839	35,934	44	227	-
IR-05	16S amplicon	100,582	44,028	44	227	-
IR-06	16S amplicon	134,272	58,575	44	402	-
IR-07	16S amplicon	100,024	35,475	35	718	-

Supplementary Table S3. Alpha diversity estimates (Chao1 richness and Shannon diversity) of groundwater microbial communities based on 16S rRNA gene sequencing. Diversity estimates are presented for each sample according to hydrological period (dry and wet). Median values for each diversity metric within each hydrological period are shown at the bottom of the table.

Sample	Season	Chao1	Shannon
BI2	wet	348.9	5.39
BI23	dry	216.5	4.06
BI262	dry	86.5	1.80
BI27	dry	127.0	1.87
BI30	dry	280.1	3.01
IR1	wet	279.9	4.36

IR3	wet	218.8	2.82
IR5	wet	227.6	3.90
IR6	wet	361.5	2.03
IR7	wet	707.2	4.90
**Median**	**Season**	**Chao1**	**Shannon**
	wet	314.4	4.13
	dry	171.75	2.44

Supplementary Table S4 – Quality metrics of metagenome-assembled genomes (MAGs). Completeness, contamination, genome size, coding density and additional genome quality metrics correspond to Chekm2 analysis and MIMAG classification was determined according to Bowers et al. (2017).

Supplementary Table S5 – Taxonomic classification of MAGs recovered based on GTDB-Tk assignments. Taxonomic assignments are reported from domain to species level according to the Genome Taxonomy Database Toolkit (GTDB-Tk).

Supplementary Table S6 – Distribution of sulfur-cycling metabolic pathways among MAGs recovered from samples BI-04, BI-06, and BI-28. MAGs were classified into functional groups according to the completeness of KEGG modules associated with dissimilatory sulfate reduction (DSR; M00596) and thiosulfate oxidation via the SOX system (M00595). Only MAGs showing 100% completeness for all required module steps were assigned to each functional category. Values indicate the number and proportion of MAGs classified as DSR-only, SOX-only, or co-encoding both pathways.

Sample	Total MAGs	DSR Only n (%)	SOX Only n (%)	Co-encoding n (%)
BI-04	77	2 (2.6%)	0 (0.0%)	2 (2.6%)
BI-06	34	6 (17.6%)	1 (2.9%)	4 (11.8%)
BI-28	74	2 (2.7%)	0 (0.0%)	0 (0.0%)

3. Conflict of interest

The authors declare that the research was conducted in the absence of any commercial or financial relationships that could be construed as a potential conflict of interest.

4. Author Contributions

Renato Romano: Conceptualization; Methodology; Investigation; Formal analysis; Data curation; Validation; Visualization; Writing – original draft; *

Tom Morita: Conceptualization; Methodology; Investigation; Formal analysis; Validation; Visualization; Project administration; Writing – original draft; *

Francielli Peres: Investigation; Methodology; Formal analysis; Data curation; Validation; Visualization; Writing – original draft; *

Rebeca Lizárraga: Investigation; Methodology; Formal analysis; Data curation; Validation; Visualization; Writing – original draft;

Lucas Padoan: Conceptualization; Methodology; Investigation; *

Maria do Nascimento: Data curation; Formal analysis; *

Livia Rocha: Investigation; *

Amanda Bendia: Resources; Validation; *

Ivo Karman: Conceptualization; Resources; Validation; Supervision; Project administration; Funding acquisition; *

Vivian Pellizari: Conceptualization; Resources; Validation; Supervision; Project administration; Funding acquisition; *
*Writing – review & editing: All authors contributed to the final revision of the manuscript.

5. Funding

This research was funded by the Chico Mendes Institute for Biodiversity Conservation – National Center for Research and Conservation of Caves (ICMBio-CECAV) in partnership with Ferro Puro Mineração, through the Technical and Scientific Cooperation Agreement (TCCE 01/2020).

6. Acknowledgments

We are deeply grateful to all the people who contributed to this work through field assistance, scientific discussions, logistical support, and friendship throughout the development of this research.

We especially thank the teams and collaborators from the Laboratory of Microbial Ecology (LECOM, University of São Paulo), the Institute of Geosciences of the University of São Paulo, and all researchers, students, technicians, and field assistants involved in sample collection, hydrochemical analyses, laboratory procedures, and discussions related to karst geomicrobiology and groundwater hydrogeochemistry.

We thank the Grupo Bambuí de Pesquisas Espeleológicas and the Meandros Espeleo Clube for providing cave maps and speleological support; Murilo Valle and Alexandre Lobo; as well as all local collaborators, cave owners, guides, and residents from the municipalities and rural communities of the Irecê Basin who assisted and welcomed the research team during field campaigns.

We are especially grateful to Claudia, posthumous guardian and guide of the Lapa Doce cave system, whose friendship, generosity, and support were fundamental during the field activities. We also

dedicate a special posthumous acknowledgment to MSc. Rosa Gamba for her guidance, friendship, mentorship, and ethical example throughout this work and beyond.

This research was supported by ICMBio-CECAV/Ferro Puro (TCCE 01/2020), with additional institutional and logistical support from the Instituto Brasileiro de Desenvolvimento e Sustentabilidade (IABS) team and collaborating laboratories and research groups.

Exclusively to assist in creating background graphical elements in Figure 8, we used artificial intelligence-based image generation tools (OpenAI, ChatGPT image generation model, accessed in 2026 via https://chat.openai.com) The scientific content, interpretation, and figure assembly were entirely performed by the authors.

7. Statements

7.1. Data Availability Statement

Raw metagenomic and 16S rRNA gene amplicon sequencing reads were deposited in the NCBI Sequence Read Archive (SRA) under BioProject accession number PRJNA1463332. High- and medium-quality metagenome-assembled genomes were deposited in Figshare under DOI 10.6084/m9.figshare.32209623.

7.2. Scope statement

This manuscript is suitable for Frontiers in Microbiology, particularly the "Microbial Life in Underexplored Aquatic Environments" special edition, because it examines microbial communities and sulfur cycling in a karst groundwater system using hydrochemical analyses, 16S rRNA gene amplicon sequencing, and genome-resolved metagenomics. The study focuses on groundwater microbial ecology, hydrochemical variability, redox gradients, and sulfur biogeochemistry in a sulfur-rich aquifer from the Irecê Basin, northeastern Brazil. The work falls within the journal's scope by addressing environmental and subsurface microbiology, microbial responses to hydrochemical conditions, and the functional potential of groundwater microbiomes in a non-hydrothermal karst aquifer.
